# Crystal engineering of nickel(ii) coordination networks sustained by aliphatic dicarboxylate linker ligands

**DOI:** 10.1039/d5ce00918a

**Published:** 2025-11-09

**Authors:** Bharti Singh, Tao He, Michael J. Zaworotko

**Affiliations:** a Department of Chemical Sciences, Bernal Institute, University of Limerick V94 T9PX Republic of Ireland xtal@ul.ie

## Abstract

Aliphatic dicarboxylate linker ligands are relatively understudied in the field of coordination networks (CNs) compared to their aromatic counterparts. Herein, we report the synthesis and characterisation of three nickel(ii) CNs comprised of mixed ditopic linkers, a linear ditopic imidazolyl ligand and three aliphatic dicarboxylates: [Ni(glu)(bimbz)], **sql-glu-Ni**, [Ni(adi)(bimbz)(H_2_O)_2_], **sql-adi-Ni**, and [Ni(muc)(bimbz)(H_2_O)]·H_2_O, **dia-muc-Ni** (bimbz = 1,4-bis-(1*H*-imidazol-1-yl)benzene, glu = glutaric acid, adi = adipic acid, muc = *trans*, *trans*-muconic acid). Single crystal X-ray diffraction studies reveal that this family of CNs is comprised from nickel-based octahedral 4-connected nodes linked through nickel-carboxylate and nickel-imidazole coordination bonds. The resulting structures can be described as non-interpenetrated square lattice, **sql**, (**sql-glu-Ni** and **sql-adi-Ni**) or 5-fold interpenetrated diamondoid, **dia**, (**dia-muc-Ni**) topology networks. A Cambridge Structural Database (CSD) mining study was conducted to evaluate the effect of node composition and structure on topology in 222 archived CNs of general formula [MLL′], [MLL′(H_2_O)], [MLL′(H_2_O)_2_], [M_2_L_2_L′] (= “pillared paddlewheel”) and 
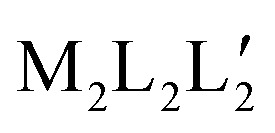
 (= “double-walled nets”) where L = aliphatic dicarboxylate linker and L′ = linear ditopic N-donor linker. In terms of prevalence, **sql**, **sql**, **neb**, **rob** and **pcu**, respectively, were found to be the most common topologies for each of these compositions. These statistics suggest that aliphatic dicarboxylate linkers can have a profound and consistent effect on the resulting topology for certain node compositions. This is especially the case for “pillared paddlewheel” nets, which favour **rob** topology over the **pcu** or “DMOF” topology that dominates for rigid linkers.

## Abstract 2

Aliphatic dicarboxylate linkers together with tailored node compositions direct Ni(ii) coordination networks toward predictable and topology specific architectures.

## Graphical abstract

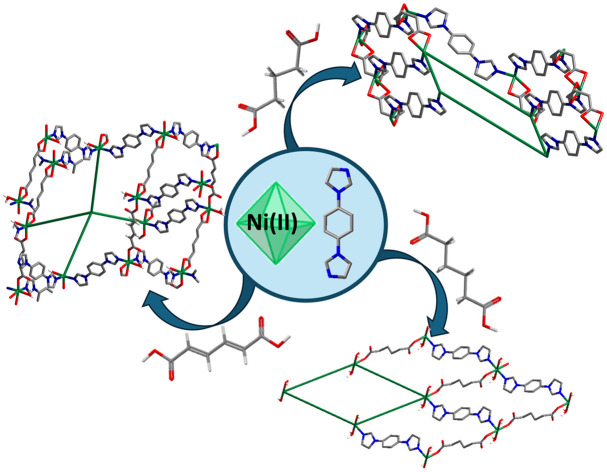

## Introduction

Crystal engineering offers well-developed approaches to systematically design families of functional crystalline materials by employing concepts such as self-assembly^1^ and “node-and-linker”^2,3^ approaches to design and construct coordination networks (CNs). Exploitation of metal clusters as molecular building blocks (MBBs)^4^ or supermolecular building blocks (SBBs)^5^ has afforded a plethora of CN families based upon low connectivity and high connectivity nodes, respectively. CNs are of particular interest to crystal engineers because they are inherently modular,^6^ meaning that systematic structure–function studies can be conducted upon families of compositionally and structurally related materials.^7–9^ The extensive properties of CNs are such that a wide range of applications, including gas/vapour storage and separation, magnetism, catalysis, optoelectronics and energy storage, are being investigated.^10–13^ This activity shows no sign of abatement with >133 737 CNs (Conquest version 2025.2.0)^14^ in the MOF subset^15^ of the Cambridge Structural Database (CSD),^16^ the majority of which are classified as potentially porous.

CNs are typically, but not always,^17,18^ comprised of metal moieties that serve as nodes and linkers that propagate the geometry of the metal or metal cluster. The most commonly employed linkers include rigid N-donors (*e.g.* 4,4′-bipyridine, 4,4′-bipy^19–22^), rigid carboxylate donors (*e.g.*, 1,4-benzenedicarboxylate, 1,4-bdc^23,24^) and rigid mixed N/O-donors (*e.g.* nicotinate^25,26^ or isonicotinate^27,28^). Interestingly, some of these prototypal CNs such as **sql** topology ELM-11^22^ and **pcu** topology nets based upon pillaring of **sql** topology CNs, *e.g.* [Cu_2_(1,4,-bdc)_2_(4,4-bipy)]^29^ and DMOF-1^24^ were amongst the first examples of flexible CNs shown to exhibit guest-induced phase transformations, a subject of growing interest with respect to functional properties.^30^

The topology of a CN provides connectivity without precise geometric and chemical details and can serve as a useful blueprint for crystal engineers to build families of related CNs once a parent CN has been identified.^6,31–33^ For example, the aforementioned linear ditopic linker ligands and 4-connected nodes are well suited to generate either **sql**^19,20^ (square lattice) or **dia**^34^ (diamondoid) topology CNs. CSD mining has revealed that extended variants of these parent linker ligands enables ligand substitution to tailor pore structure and properties with retention of topology.^7,35^ Likewise, as detailed herein, it is feasible to replace a pyridyl moiety with an imidazolyl group, with 1,4-bis(imidazol-1-yl) benzene (bimbz) being an example of such an N-donor linker that can be used on its own^36^ or as part of a mixed linker ligand system.^37^ Dicarboxylate linkers also offer diversity and can enable fine-tuning of CN structure and properties^38,39^ although aliphatic dicarboxylates, which have the potential to enhance flexibility of CNs,^40,41^ remain understudied compared to their aromatic counterparts (Scheme S1a). A key effect of ligand flexibility is that multiple topologies can exist for the same molecular building blocks, resulting in supramolecular isomers.^42^ Aliphatic linkers such as glutaric acid (glu) and adipic acid (adi) possess intrinsic conformational flexibility (*gauche*–*gauche*, *gauche*–*anti*, and *anti*–*anti*), while muconic acid (muc) can adopt both *cis* and *trans* conformations, offering conformational flexibility in CNs.^43^ In contrast, aromatic linkers *e.g.* 1,4-bdc are rigid and do not contribute directly to framework flexibility. For instance, the flexibility observed in MIL-53 (based on 1,4-bdc) arises from the butterfly-type motion of the metal nodes.^44^ Overall, while aliphatic linkers can enable network flexibility, rigid aromatic linkers can also yield flexible CNs *via* alternative mechanisms.^30^

In this study, we report the structures of three CNs prepared from Ni(ii), bimbz and three different aliphatic dicarboxylic acids: glutaric acid (H_2_glu), adipic acid (H_2_adi) and *trans*, *trans*-muconic acid (H_2_muc) as well as a CSD database mining study of the topologies exhibited by aliphatic dicarboxylate linkers and imidazole-based N-donor linkers and these three acids (H_2_glu, 24 hits; H_2_adi, 26 hits; H_2_muc, 0 hits, Scheme S1a and Table S1). We selected Ni(ii) for this study as it more reliably forms monometallic MN_2_(CO_2_)_2_ molecular building blocks compared to later transition metals, which have a greater tendency to exhibit bimetallic structures.

## Experimental

### Materials and instrumentation

The linker 1,4-bis-(1*H*-imidazol-1-yl) benzene was synthesized with a modification (SI) of the reported procedure.^45^ Other reagents and solvents are commercially available and were used without further purification. As detailed below, three CNs were prepared by reaction of NiNO_3_·6H_2_O, bimbz and an aliphatic dicarboxylic acid to afford [Ni(glu)(bimbz)] (**sql-glu-Ni**), [Ni(adi)(bimbz)(H_2_O)_2_] (**sql-adi-Ni**) and [Ni(muc)(bimbz)(H_2_O)]·H_2_O (**dia-muc-Ni**).

#### [Ni(glu)(bimbz)] (**sql-glu-Ni**)

NiNO_3_·6H_2_O (0.1 mmol, 29.07 mg), H_2_glu (0.1 mmol, 13.21 mg) and bimbz (0.1 mmol, 21.02 mg) were dissolved in 8 mL deionised water at pH = 8 controlled by addition of 0.1 M NaOH and stirred for 15 min. The reaction mixture was placed in a Teflon container and heated at 160 °C for 3 days before cooling to room temperature. Light green block-shaped crystals were harvested, washed with water, and air dried (yield = 8.58 mg, ∼65% based on H_2_glu).

#### [Ni(adi)(bimbz)(H_2_O)_2_] (**sql-adi-Ni**)

NiNO_3_·6H_2_O (0.1 mmol, 29.07 mg), H_2_adi (0.1 mmol, 14.61 mg) and bimbz (0.1 mmol, 21.02 mg) were dissolved in 8 mL of deionised water at pH = 8 controlled by addition of by 0.1 M NaOH and stirred for 15 min. The reaction mixture was placed in a Teflon container and reacted at 160 °C for 3 days before cooling to room temperature. Light blue plate-shaped crystals were obtained, washed with water, and air dried (yield = 10.08 mg, ∼69% based on H_2_adi).

#### [Ni(muc)(bimbz)(H_2_O)]·H_2_O (**dia-muc-Ni**)

NiNO_3_·6H_2_O (0.1 mmol, 29.07 mg), H_2_muc (0.1 mmol, 14.21 mg) and bimbz (0.1 mmol, 21.02 mg) were dissolved in 8 mL deionised water at pH = 8 controlled by 0.1 M NaOH, then stirred for 15 min. The reaction mixture was placed in a Teflon container and reacted at 160 °C for 3 days before cooling to room temperature. Light green block-shaped crystals were obtained, washed with water, and air dried (yield = 8.95 mg, ∼63% based on H_2_muc).

### Physical measurements

Crystal structures were determined by single crystal X-ray diffraction (SCXRD) with MoKα radiation (*λ* = 0.71073 Å) on a Bruker D8 Quest fixed-chi diffractometer equipped with a Photon II detector and a nitrogen-flow Oxford Cryosystem attachment. Frames were collected at low temperature by *ω*, *φ*, and 2*θ* rotation at 10 s per frame using SAINT. Data was indexed, integrated, and scaled in APEX4.^46,47^ Absorption corrections were performed by the multi-scan method using SADABS.^47^ Space groups were determined using XPREP incorporated within APEX4. Structure solution, refinement, and data output were carried out using OLEX 2 with the inbuilt SHELXTL-2018 program.^48^ Non-hydrogen atoms were refined anisotropically. Hydrogen atoms were placed using molecular geometry at idealised locations and assigned isotropic thermal parameters depending on the equivalent displacement parameters of their carriers. Images were created using the DIAMOND program.^49^ Hydrogen bonding interactions in the crystal lattice were calculated using SHELXTL and DIAMOND. Crystal data and structure refinement parameters for **sql-glu-Ni**, **sql-adi-Ni** and **dia-muc-Ni** are presented in Table S2. Crystallographic data have been deposited in the CSD (CCDC 2449933–2449935). In **sql-glu-Ni**, electron density consistent with H_2_O at occupancy 0.16 was observed.

Powder X-ray diffraction (PXRD) experiments were conducted using microcrystalline samples on a PANalytical Empyrean diffractometer equipped with PIXcel3D detector (40 kV; 40 mA; CuKα_1,2_*λ* = 1.5418 Å) in Bragg–Brentano geometry. Data were collected with a step size of 0.05 at a count time of 1 s per step over the range 5° < 2*θ* < 40°. PXRD patterns were also calculated from SCXRD structures using Mercury software. The experimental and calculated PXRD patterns are in good agreement suggested that the bulk samples are in a single phase (Fig. S1). Thermogravimetric analyses were performed under N_2_ flow using a TA Instruments Q50 system. A sample was loaded into an aluminium sample pan and heated at 5 °C min^−1^ from room temperature to 500 °C. Fourier Transform Infrared (FTIR) spectra were collected on a PerkinElmer Spectrum 100 spectrometer with Universal ATR accessory from 600 cm^−1^ to 4000 cm^−1^ (Fig. S3).

## Result and discussion

**Scheme 1:**
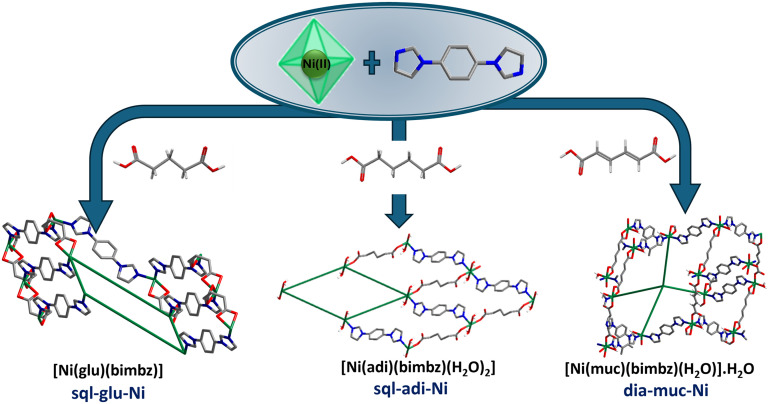
The three CNs isolated herein, all of which have the formula [Ni(L)(bimbz)]_*n*_, L = aliphatic dicarboxylate.

Hydrothermal reaction of NiNO_3_·6H_2_O, bimbz and an aliphatic dicarboxylic acid afforded three new CNs, [Ni(glu)(bimbz)] (**sql-glu-Ni**), [Ni(adi)(bimbz)(H_2_O)_2_] (**sql-adi-Ni**) and [Ni(muc)(bimbz)(H_2_O)]·H_2_O (**dia-muc-Ni**) (Scheme 1).

### [Ni(glu)(bimbz)], **sql-glu-Ni**

**Fig. 1:**
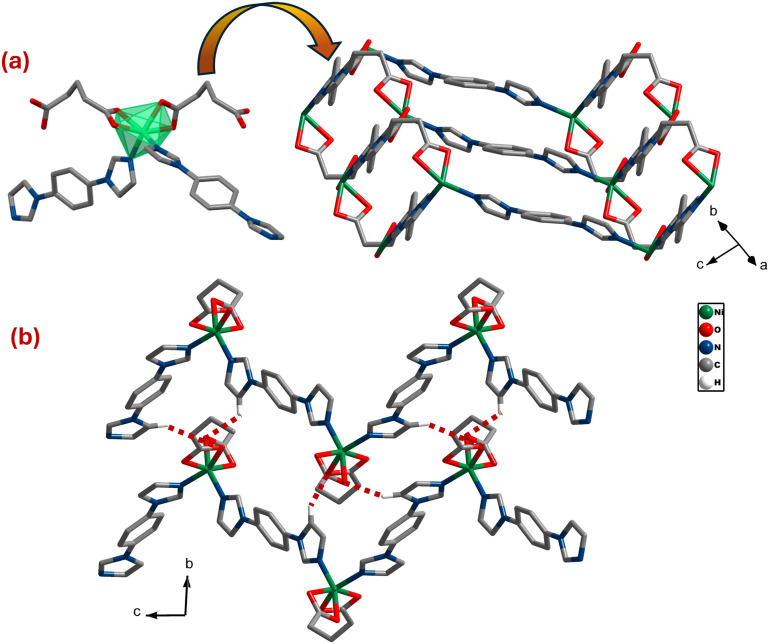
(a) Distorted {NiN_2_O_4_} octahedra connected by glu and bimbz linkers form 2D zigzag sheets (b) C–H⋯O_(COOH)_ interactions (red dotted lines) between a carboxylate of glu in one sheet to an imidazole of bimbz from an adjacent sheet. Hydrogen atoms omitted for clarity.

Single crystal X-ray diffraction (SCXRD) revealed that **sql-glu-Ni** had crystallized in triclinic space group *P*1̄. The asymmetric unit contains one crystallographically independent Ni(ii), two halves of bimbz ligands and one glu ligand. In **sql-glu-Ni**, Ni(ii) adopts distorted octahedral {NiO_4_N_2_} geometry with four oxygens from two carboxylates of two different glu ligands wherein carboxylate groups bind to Ni(ii) *via* the (κ^2^)-(κ^2^)-μ_2_ mode (Scheme S2a). The remaining two coordination sites are occupied by N-donors from bimbz ligands. The bond lengths (Ni–O) and bond angles (∠O–Ni–O) fall within the expected ranges (Table S3).^50,51^ As shown in Fig. 1a, the distorted octahedra are connected by glu linkers to form 1D chains along the *a*-axis which are in turn connected by bimbz linkers to form 2D zig-zag sheets with **sql** topology. These sheets are further connected by nonbonding C–H⋯O interactions (C⋯O, 3.218 Å) between the carboxylate group of a glu in one sheet to an imidazole ring from an adjacent sheet (Fig. 1b). A CSD survey indicates that only two structures are previously reported comprising glu and bimbz: [Cd_2_(glu)_2_(bimbz)]^52^ (CSD refcode, GIRCER), having 2D cadmium-glu sheets pillared by bimbz linkers that form a three-dimensional (3D) architecture; [Zn(glu)(bimbz)]·H_2_O ^53^ (CSD refcode, HEWMOP) which is related to **sql-glu-Ni**.

### [Ni(adi)(bimbz)(H_2_O)_2_], **sql-adi-Ni**

**Fig. 2:**
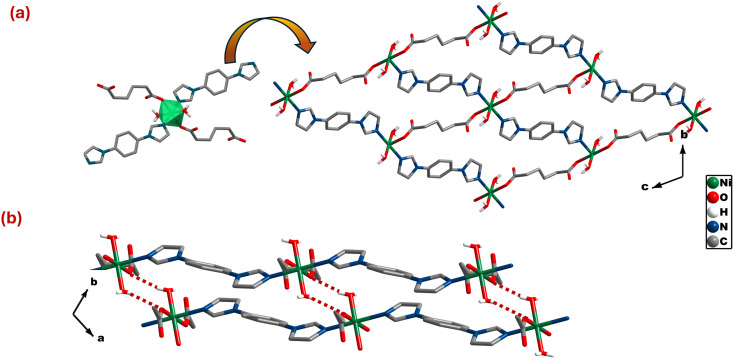
(a) Distorted {NiN_2_O_4_} octahedra are connected by adi and bimbz linkers to form 2D **sql** sheets (b) O–H⋯O_(COOH)_ interactions (red dotted lines) exist between aqua ligands and carboxylate moieties of adi ligands from an adjacent sheet. Hydrogen atoms omitted for clarity.

**sql-adi-Ni** crystallized in triclinic space group *P*1̄ with a asymmetric unit comprising one crystallographically independent Ni(ii), half of adi and bimbz, and one water molecule. The coordination environment of **sql-adi-Ni** comprises two bimbz ligands coordinated through nitrogen atoms in *trans* configuration and carboxylates of two adi ligands. The remaining two apical coordination sites are occupied by aqua ligands. In **sql-adi-Ni**, the carboxylate group of the adi ligand binds in a monodentate manner (κ^1^)-(κ^1^)-μ_2_ (Scheme S2b). The bond lengths (Ni–O) and bond angles (∠O–Ni–O) fall within expected ranges (Table S3).^54,55^ As shown in Fig. 2a, {NiO_4_N_2_} octahedra are linked by adi ligands to from 1D chains, which are cross-linked by bimbz ligands to form 2D sheets with **sql** topology. These 2D sheets are cross-linked by H-bonding, O–H⋯O (O⋯O, 2.870 Å), between the apical aqua ligands and carboxylate oxygens of adi ligands from an adjacent sheet (Fig. 2b). A CSD analysis revealed only one hit involving adi and bimbz, [Cu(adi)(bimbz)(H_2_O)_2_]^56^ (CSD refcode, PILHUP). PILHUP is structurally related to **sql-adi-Ni**; both exhibit **sql** topology but with void volumes of 0% and 21.4% for **sql-adi-Ni** and PILHUP, respectively (probe radius: 1.2 Å and approx. grid spacing: 0.3 Å).

### [Ni(muc)(bimbz)(H_2_O)]·H_2_O **dia-muc-Ni**

**Fig. 3:**
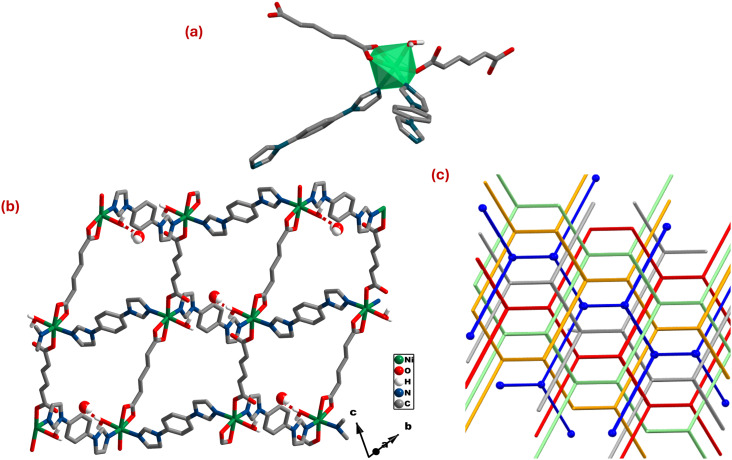
(a) Distorted {NiN_2_O_4_} octahedra are connected by muc and bimbz linkers to form (b) a **dia** topology CN with O–H⋯O_(COOH)_ hydrogen bonds (dotted lines) between the lattice H_2_O molecules and carboxylate oxygens of muc. (c) 5-fold interpenetration is exhibited by **dia-muc-Ni**. Hydrogen atoms omitted for clarity.

**dia-muc-Ni** crystallized in triclinic space group *P*1̄ with an asymmetric unit comprising one crystallographically independent Ni(ii), two halves of muc and bimbz ligands, one aqua ligand and one lattice H_2_O molecule. In **dia-muc-Ni**, Ni(ii) adopted distorted octahedral geometry with two coordination sites occupied by nitrogen atoms from different bimbz ligands and two sites occupied by three carboxylate oxygens of two muc ligands *via* (κ^1^)-(κ^1^)-μ_2_ and (κ^2^)-(κ^2^)-μ_2_ binding modes (Scheme S2c and d). The remaining coordination sites are occupied by aqua ligands. The bond lengths (Ni–O) and bond angles (∠O–Ni–O) lie within expected ranges (Table S3).^57^ In **dia-muc-Ni**, distorted {NiN_2_O_4_} octahedra are linked by linear bimbz and muc ligands to form a 3D CN with **dia** topology and 5-fold interpenetration (Fig. 3b and c). There are no CSD entries involving muc and bimbz.

The phase purity of **sql-glu-Ni**, **sql-adi-Ni** and **dia-muc-Ni** was examined by PXRD and found to be single phase (Fig. S1). We addressed the hydrolytic stability of these solids by exposing powder samples of **sql-glu-Ni**, **sql-adi-Ni** and **dia-muc-Ni** to 75% RH at 40 °C for one week in a humidity chamber. No phase changes were observed (Fig. S1).

### Thermal analysis

**Fig. 4:**
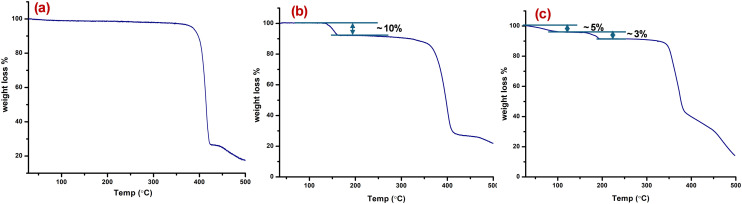
Thermogravimetric profiles of (a) **sql-glu-Ni** (b) **sql-adi-Ni** and (c) **dia-muc-Ni**.

Thermogravimetric analysis profiles are presented in Fig. 4. **sql-glu-Ni** showed negligible weight loss up to 400 °C. **sql-adi-Ni** exhibited ∼10% weight loss, consistent with loss of the aqua ligands; PXRD revealed that it became amorphous before reverting to crystallinity after exposure to water vapour (Fig. S2). The thermal properties of **dia-muc-Ni** reveal a two-step weight loss, consistent with loss of lattice water (∼5%), before a weight loss (∼3%) corresponding to aqua ligands and then show thermal stability to 380 °C.

### Topological distribution in aliphatic dicarboxylate linked CNs

**Fig. 5:**
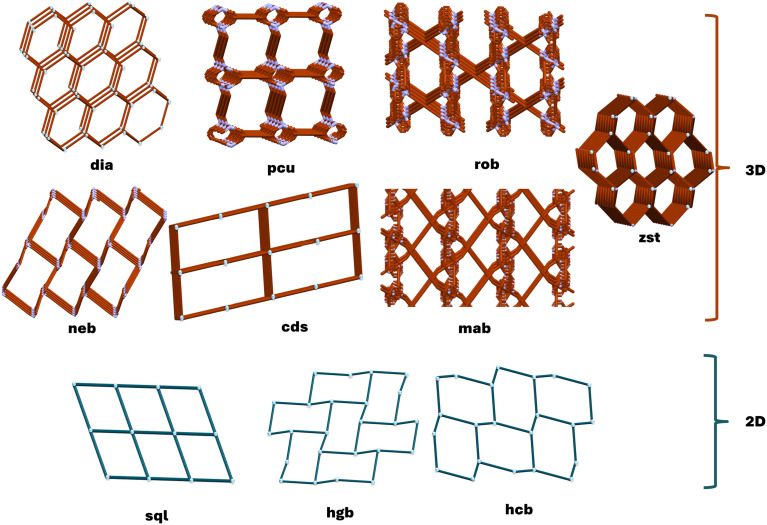
A subset of 222 CNs comprising aliphatic dicarboxylate and N-donor linkers revealed **dia**, **pcu**, **rob**, **neb**, **cds**, **mab**, **zst** (3D) and **sql**, **hcb**, **hgb** (2D) topology nets.

An analysis of compositionally and structurally similar CNs can provide insight into how linkers impact topology. We therefore conducted CSD database mining using ConQuest^14^ software to analyse CNs comprising the three aliphatic acids studied herein glutaric acid (glu), adipic acid (adi) and *trans*-, *trans*-muconic acid (muc). Analysis of the results revealed that the five most common node compositions have general formulae [MLL′], [MLL′(H_2_O)], [MLL′(H_2_O)_2_], [M_2_L_2_L′] and 
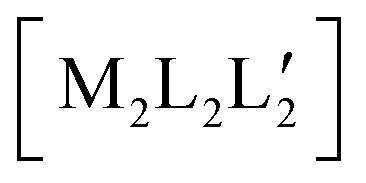
 where M = divalent metal, L = glu, adi or muc linker and L′ = N-donor linker (imidazolyl or 4-pyridyl linker). Topology determination was conducted using ToposPro^58^ software. Among the 222 hits in this subset (see Table S1), [MLL′], [M_2_L_2_L′] and 
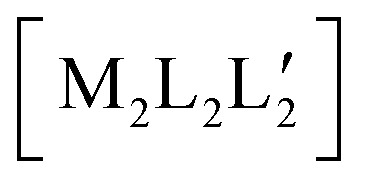
 were the most commonly observed compositions with 40, 35 and 37 entries, respectively. [MLL′(H_2_O)] and [MLL′(H_2_O)_2_] had 14 and 11 entries, respectively. Specific topologies included **dia**, **pcu**, **rob**, **neb**, **cds**, **mab**, **zst** (3D) and **sql**, **hcb**, **hgb** (2D) (Fig. 5).

**Fig. 6:**
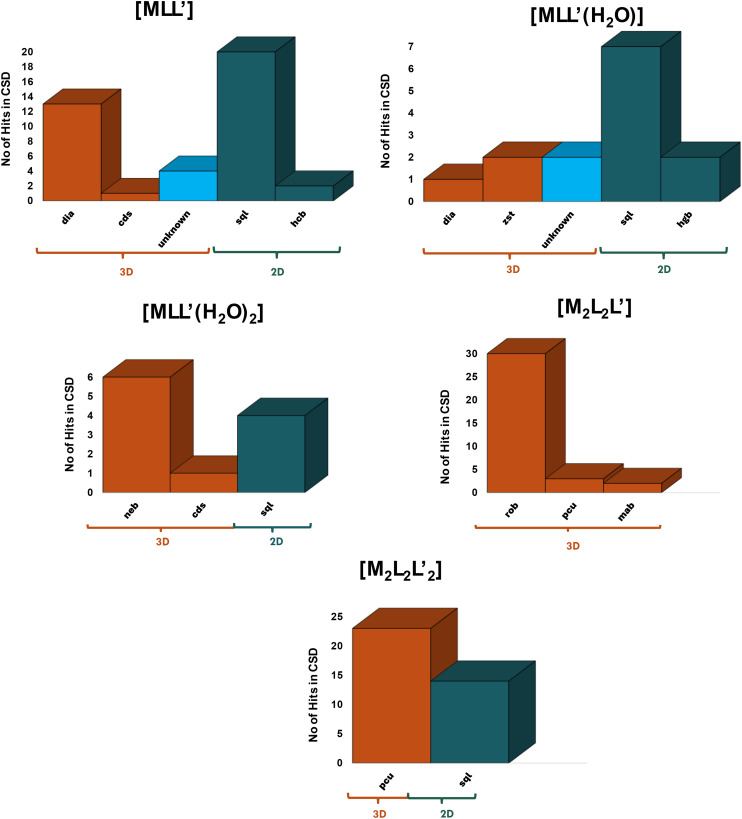
Histograms representing statistics of topological distributions present in node compositions [MLL′], [MLL′(H_2_O)], [MLL′(H_2_O)_2_], [M_2_L_2_L′], 
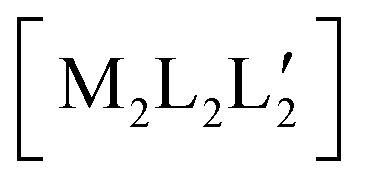
.

**Fig. 7:**
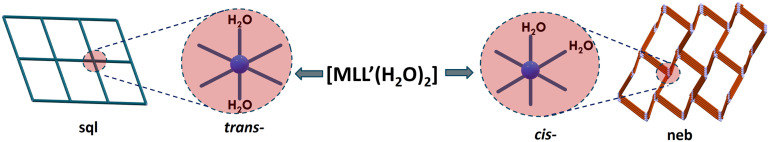
The effect of node geometry on resulting topology in [MLL′(H_2_O)_2_] (*cis*-, **neb** and *trans*-, **sql**).

Fig. 6 details the topology distribution for the five most common node compositions. [MLL′] nodes were found to form **sql** networks most frequently (20 hits), **dia** being the second most prevalent topology (13 hits) with **cds** and **hcb** topologies also found (Fig. 6). For [MLL′(H_2_O)], two additional topologies were observed, **zst** and **hgb**, but **sql** remained the most common topology (7 hits). For [MLL′(H_2_O)_2_], 3D nets such as **neb** (most prevalent) and **cds** were observed along with **sql** topology. All nets with node compositions [MLL′], [MLL′(H_2_O)] and [MLL′(H_2_O)_2_] comprise 4-connected nodes and mononuclear MBBs. Whereas **sql** was most commonly found for [MLL′] and [MLL′(H_2_O)] nodes. **sql** was also the most commonly observed topology but for [MLL′(H_2_O)_2_] the 3D topology **neb** was found to be prevalent. Analysis of the coordination geometries in the **neb** structures revealed that the aqua ligands adopt *cis*-geometry, which would mitigate against **sql** topology (Fig. 7).

**Fig. 8:**
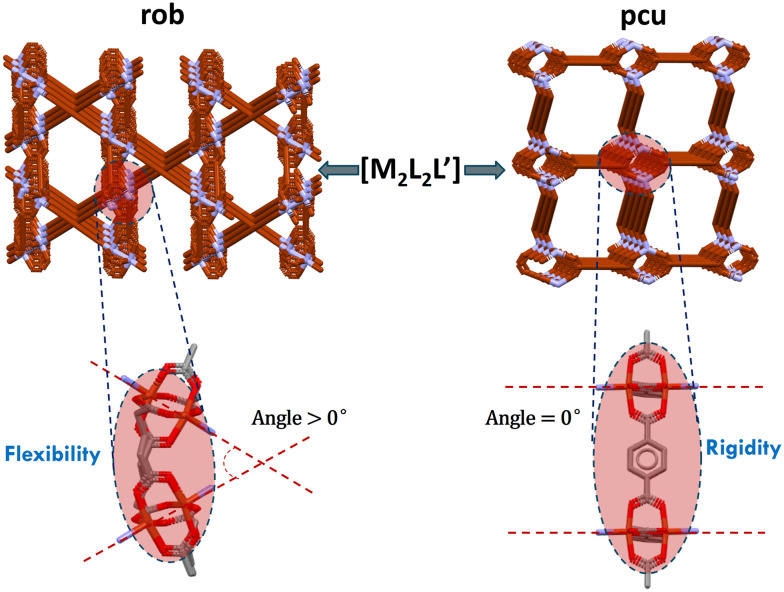
Illustration of the spatial arrangements of paddlewheel nodes in [M_2_L_2_L′] CNs corresponding to **rob** and **pcu** topologies.


**Fig. 9:**
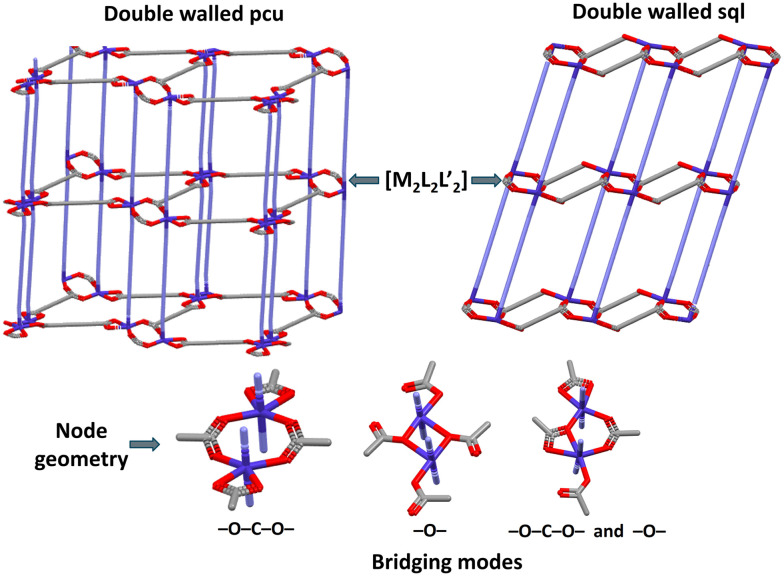
Illustration of double-walled **pcu** and **sql** topologies and different node structures in 
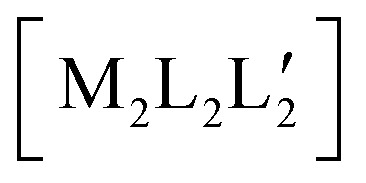
.

Interestingly, for [M_2_L_2_L′] nodes, the 3D **rob** topology was found to be dominant with 30 of 35 entries; **pcu** and **mab** 3D nets were also present. All of the **rob** entries are comprised of “paddlewheel” MBBs and this raises the issue of why the much more commonly encountered **pcu** topology is hardly observed in this subset of structures. Our analysis indicates that the conformational flexibility of aliphatic dicarboxylate ligands permits rotation of the paddlewheel nodes, thereby facilitating the formation of **rob** rather than **pcu** topology. In contrast, the intrinsic rigidity of aromatic dicarboxylate ligands restricts such rotational freedom, impeding the adoption of the same topology (Fig. 8). 3/35 **pcu** topology nets can be classified as double-walled **pcu** (Fig. 6) with the paddlewheel or corner sharing nodes. For example, FIGQUJ^59^ comprises a paddlewheel whereas TOTTUU^60^ has a corner-sharing arrangement. 2/35 **mab** topology nets can be interpreted as a special case of **rob** topology wherein the length of the rigid linker promotes coordination to the second adjacent node located opposite the paddlewheel node.

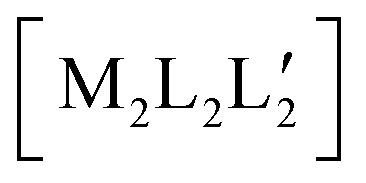
 is the second most common node composition with 37 refcodes, with all entries exhibiting **pcu** (23 entries) or **sql** topology (14 entries) (Fig. 6). All **pcu** nets are double-walled, with nodes comprised of carboxylate bridged metal centres, either though –O–C–O– (19/24), –O– (edge sharing, 1/24), or both modes simultaneously (3/24), as exemplified by QOHTOX,^61^ LECBOL^62^ and TOTTOO^60^ respectively. The **sql**-topology refcodes are also double-walled, where the node structure is same as the **pcu** topology refcodes, metal centres bridged by –O–C–O– or –O– carboxylate moieties, *e.g.* AYALUH^63^ or GINWOQ^64^ and IBOVEE^65^ or NOWZAD,^66^ respectively. The node geometry (Fig. 9) is consistent with the formation of double-walled **pcu** and **sql** CNs, diverging from more common single-walled variants which predominantly consist of mononuclear [MLL′] and paddlewheel dimer nodes [M_2_L_2_L′].

**sql-glu-Ni**, **sql-adi-Ni** and **dia-muc-Ni** are comprised of [MLL′], [MLL′(H_2_O)_2_] and [MLL′(H_2_O)] nodes, respectively. **sql-glu-Ni** and **sql-adi-Ni** adopted **sql** topology as expected based upon their node compositions as this is the most common topology for their respective node compositions. **dia-muc-Ni** is a rare example of **dia** topology for the [MLL′(H_2_O)] node composition, being only the second such example.

Ligand length tends to be more important than flexibility when it comes to enabling interpenetration. Among the analysed 222 CNs (Table S1), interpenetration was observed as follows: 8 glu (12%); 21 adi (51%); 23 muc (88%) (Fig. S4). As expected, the shorter linker, glu, favours non-interpenetrated CNs, whereas muc tends towards interpenetrated structures. These trends align with our results: **sql-glu-Ni** and **sql-adi-Ni** are non-interpenetrated; **dia-muc-Ni** is 5-fold interpenetrated.

Overall, when the structures of **sql-glu-Ni**, **sql-adi-Ni**, and **dia-muc-Ni** are put into the context of our CSD survey, we see a key crystal engineering message: node composition and configuration can critically impact the resulting structures but in a reasonably predictable manner. In the structures reported herein, even though the same synthesis conditions were used to prepare **sql-glu-Ni**, **sql-adi-Ni**, and **dia-muc-Ni**, differing only in the aliphatic acid used, their node compositions, even though all mononuclear, were observed to be different. For the other two compositions typically seen for aliphatic dicarboxylate linkers and N-donor linkers, very different outcomes are to be expected based upon CSD statistics. Specifically, [M_2_L_2_L′] and 
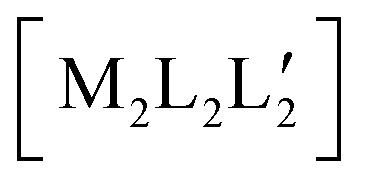
 node topologies, which can presumably be driven by stoichiometry, tend to result in otherwise rare **rob** single-walled topology networks or “double-walled” **pcu** or **sql** topology CNs. Further, for these metal dimer MBBs, the resulting topologies are controlled by the node geometry and/or the undulating nature of the network structure.

## Conclusions

The new structures reported herein, when placed in the context of a topology survey of all CNs in the CSD involving glu, adi, and muc ligands, offer insight into the factors that can govern a specific topology. This study conveys three main crystal engineering insights: (i) node composition and configuration can critically impact the resulting structures but in a reasonably predictable manner; (ii) node compositions can be driven by stoichiometry that may in turn result in different topologies; (iii) resulting topologies may arise from a synergistic interplay between the coordination geometry of the nodes and the inherent undulating nature of the network structure. The present study underscores key considerations for the rational design and construction of CNs with desired topological architectures including rare topologies such as **rob**.

## Author contributions

The manuscript was written through contributions of all authors. All authors have given approval to the final version of the manuscript. CRediT: B. S. and T. H.: conceptualization, investigation, methodology, writing – original draft, review and editing; M. J. Z.: funding acquisition, formal analysis, writing – review and editing.

## Conflicts of interest

There are no conflicts to declare.

## Supplementary Material

CE-027-D5CE00918A-s001

CE-027-D5CE00918A-s002

## Data Availability

The data supporting the findings of this study are available in the supplementary information (SI) or from the authors upon request. Supplementary information is available. See DOI: https://doi.org/10.1039/d5ce00918a. CCDC 2449933–2449935 contain the supplementary crystallographic data for this paper.^67*a*–*c*^
