# Supplementary material for: Crystal engineering of nickel(ii) coordination networks sustained by aliphatic dicarboxylate linker ligands
Source: CrystEngComm. 2025 Nov 9;27(48):7865–74. doi: 10.1039/d5ce00918a (PMC12626055; doi:10.1039/d5ce00918a)
Supplement: CE-027-D5CE00918A-s001 [file CE-027-D5CE00918A-s001.pdf]

Supplementary Information (SI) for CrystEngComm.
This journal is © The Royal Society of Chemistry 2025

Supplementary Information

Crystal engineering of Nickel(II) coordination networks sustained by aliphatic dicarboxylate linker ligands

Bharti Singh, Tao He and Micheal J. Zaworotko

Department of Chemical Sciences, Bernal Institute, University of Limerick, V94 T9PX, Republic of Ireland

E-mail: xtal@ul.ie

Contents

1. CSD survey……………………………………………………………………………2

2. Methods (Ligand synthesis)…………………………………………………………….15

2. Crystallographic information table……………………………………………...……....16

3. PXRD……………………………………………………………………………...17

4. Coordination modes of aliphatic ligands………………………………………………..18

3. FTIR spectra……………………...…………………………………………………..19

5. Selected bond lengths (Å) and angles (°) for solids………………………………………19

1. CSD Survey

A survey on number of CNs present in CSD with selected aliphatic and aromatic dicarboxylate, shows that aliphatic dicarboxylate are understudied in comparison to aromatic dicarboxylates. Scheme S1 show that comparative histogram with some examples of aliphatic dicarboxylates, like Glutaric acid (H_2glu), Adipic acid (H_2adi), and Muconic acid (H_2muc) and most celebrated aromatic dicarboxylates, 1,4-benzene dicarboxylic acid (H_2bdc), 1,4-biphenly dicarboxylic acid (H_2bpdc) and 2,6-naphthalene dicarboxylic acid(H_2ndc).

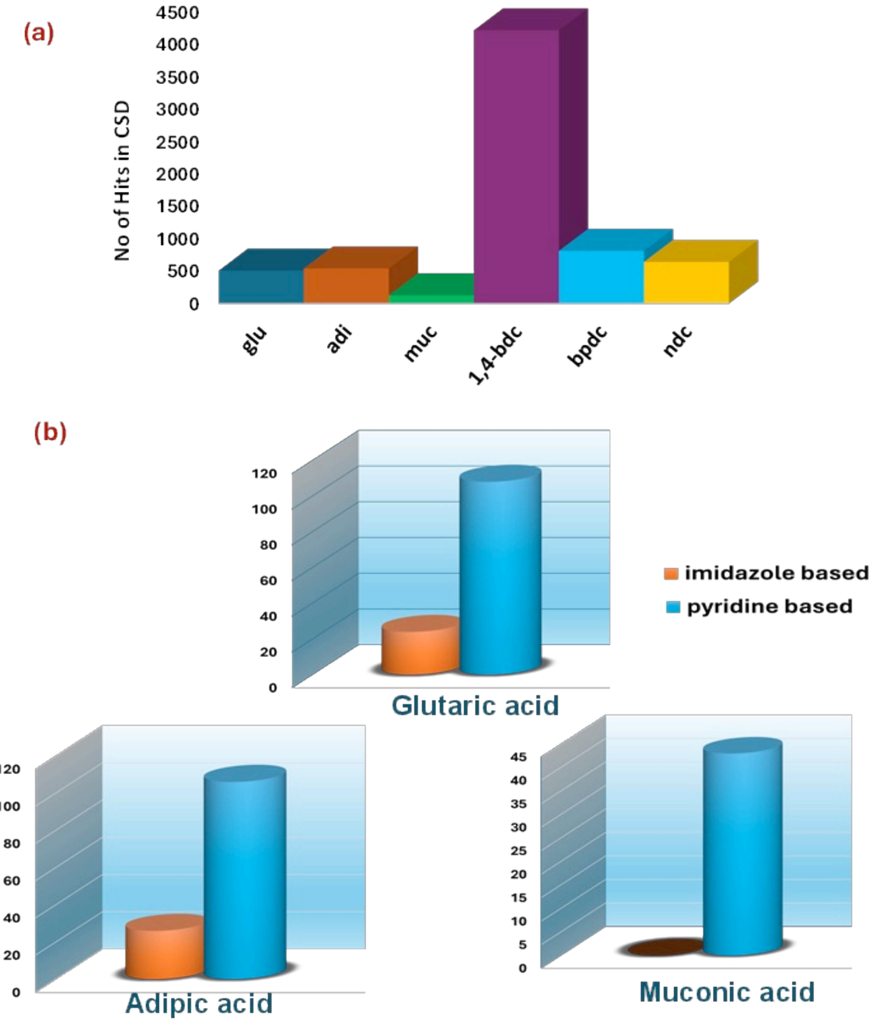

Scheme S1 (a) Histogram view of number of CNs in CSD with aliphatic and aromatic dicarboxylates **(b)** Histogram representing the number of CNs in CSD with glu, adi and muc

Table S1 Summary of node compositions, topologies, node geometries, and binding modes of aliphatic dicarboxylate ligands (glu, adi, and muc) in (CNs) as reported in the literature.

Node composition	CSD refcode	Solid	MBB/RBB	Binding modes of dicarboxylates	Topology/ point symbol	Interpenetrate d /non-interpenetrated	Gas adsorption studies
**[MLL']**	FEPVEE	[Zn(glu)(edpc)][1]	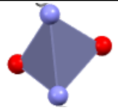 Mononulcear	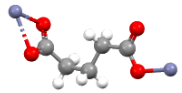 $(\kappa2)$-(κ^1)-μ_2	sql	2-fold interpenetration	Not reported
**[MLL']**	LIBJIS	[In(Glu)(bipy)(HGlu)][2]	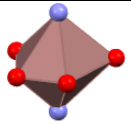 Mononulcear	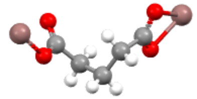 (κ^1)-(κ^2)-μ_2	sql	No-interpenetration	Not reported
**[MLL']**	GIPXIO	[Zn(glu)(bpe)][3]	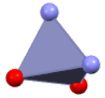 Mononulcear	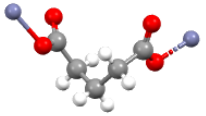 (κ^1)-(κ^1)-μ_2	sql	No-interpenetration	Stepped CO_2 isotherm was reported
**[MLL']**	SICCEO	[Zn(glu)(dpb)][4]	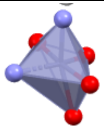 Mononulcear	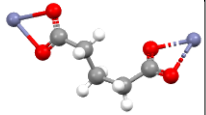 (κ^2)-(κ^2)-μ_2	sql	No-interpenetration	Not reported
**[MLL']**	ZERJAJ	[Zn(glu)(bps)][5]	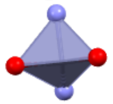 Mononulcear	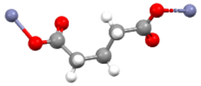 (κ^1)-(κ^1)-μ_2	sql	No-interpenetration	Not reported
**[MLL']**	KOQJOR	[Zn(glu)(4-bpmp)]·$6H_2O$[6]	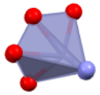 Mononulcear	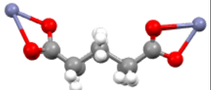 (κ^2)-(κ^2)-μ_2	dia	2-fold interpenetration	Not reported

**[MLL']**	HOPZOC	[Co(glu)(dpa)][7]	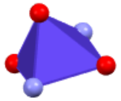 Mononulcear	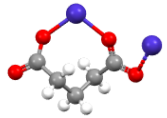 (κ^1)- $(\kappa^1$- $\kappa^1)$-μ_4	$(4.6^2)(4.6^5.8^4)$	No-interpenetration	Not reported
**[MLL']**	HOPZUI	[Ni(glu)(dpa)][7]	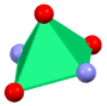 Mononulcear	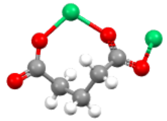 (κ^1)- $(\kappa^1$- $\kappa^1)$-μ_4	$(4.6^2)(4.6^5.8^4)$	No-interpenetration	Not reported
**[MLL']**	HOQBAR	[Cu(glu)(dpa)][7]	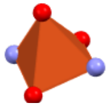 Mononulcear	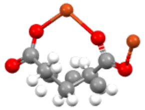 (κ^1)- $(\kappa^1$- $\kappa^1)$-μ_4	$(4.6^2)(4.6^5.8^4)$	No-interpenetration	Not reported
**[MLL']**	FOLBOZ	[Zn(adi)(BPHY)[8]	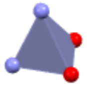 Mononulcear	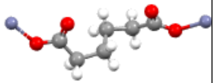 (κ^1)-(κ^1)-μ_2	sql	No-interpenetration	Not reported
**[MLL']**	FUHMAY	[Cu(adi)(3-pina)][9]	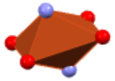 Mononulcear	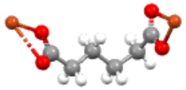 (κ^2)-(κ^2)-μ_2	sql	No-interpenetration	Not reported
**[MLL']**	NABMUZ	[Cu(adi)(bipy)][10]	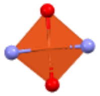 Mononulcear	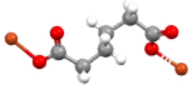 (κ^1)-(κ^1)-μ_2	sql	No-interpenetration	Not reported
**[MLL']**	NUXZEM	[Cu(adi)(bpt)][11]	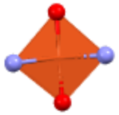 Mononulcear	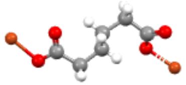 (κ^1)-(κ^1)-μ_2	sql	2-fold Interpenetration	Not reported
**[MLL']**	ZERJEN	[Zn(adi)(bps)][5]	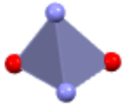 Mononulcear	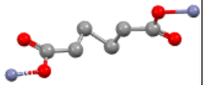 (κ^1)-(κ^1)-μ_2	sql	No-interpenetration	Not reported

**[MLL']**	KOQKIM	[Zn(adp)(4-bpmp)][6]	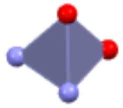 Mononulcear	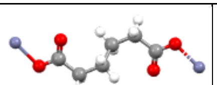 (κ^1)-(κ^1)-μ_2	dia	2-fold interpenetrated	Not reported
**[MLL']**	OVEDAV	[Cd(adi)(bpmp)][12]	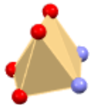 Mononulcear	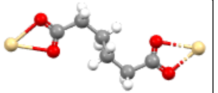 (κ^2)-(κ^2)-μ_2	dia	5-fold interpenetration	Not reported
**[MLL']**	VIXVUV	[Zn(adi)(bpa)][13]	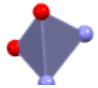 Mononulcear	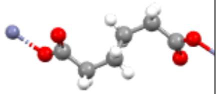 (κ^1)-(κ^1)-μ_2	dia	4-fold interpenetration	Exhibited an S-shape CO_2 adsorption isotherm at 196 K
**[MLL']**	LIPJUQ	[Zn(adp)(dpa)]· H_2O[14]	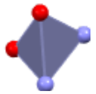 Mononulcear	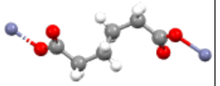 (κ^1)-(κ^1)-μ_2	hcb	2-fold interpenetration	Not reported
**[MLL']**	QIWBAB	[Zn(muc)(azobp)][15]	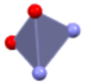 Mononulcear	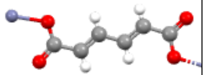 (κ^1)-(κ^1)-μ_2	sql	2-fold interpenetration	Not reported
**[MLL']**	BUVPIT	[Zn(muc)(bipy)][16]	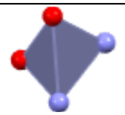 Mononulcear	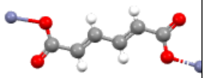 (κ^1)-(κ^1)-μ_2	dia	5-fold interpenetration	Not reported
**[MLL']**	EBOBUV	[Zn(muc)(bpe)][17]	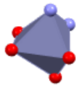 Mononulcear	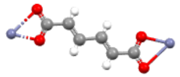 (κ^2)-(κ^2)-μ_2	dia	4-fold interpenetration	N_2, H_2, CO_2 isotherms are reported
**[MLL']**	EBOCOQ	[Zn(muc)(bpe)]. I_2[17]	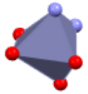 Mononulcear	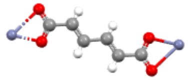 (κ^2)-(κ^2)-μ_2	dia	4-fold interpenetration	N_2, H_2, CO_2 isotherm are reported

**[MLL']**	QIVYUR	[Zn(muc)(bpe)]. H_2O[18]	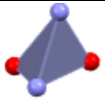 Mononulcear	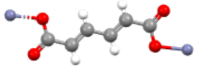 (κ^1)-(κ^1)-μ_2	dia	5-fold interpenetration	Not reported
**[MLL']**	XUJREB	[Zn(muc)(1,4-bpetmb)][19]	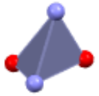 Mononulcear	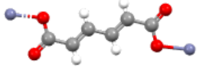 (κ^1)-(κ^1)-μ_2	dia	6-fold interpenetration	Not reported
**[MLL']**	VIXVEF	[Zn(muc)(bpa)]. $2H_2O$[13]	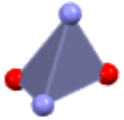 Mononulcear	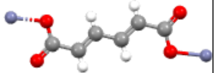 (κ^1)-(κ^1)-μ_2	dia	4-fold interpenetration	exhibited an S-shape CO_2 adsorption isotherm at 196 K
**[MLL']**	OQUGEP	[Co(bib)(glu)].2 H_2O[20]	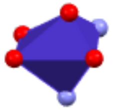 Mononulcear	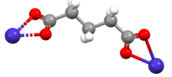 (κ^2)-(κ^2)-μ_2	sql	Non-interpenetrated	Not reported
**[MLL']**	PAMVAC	[Zn(bib)(glu)].4 H_2O[21]	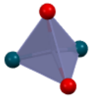 Mononulcear	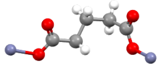 (κ^1)-(κ^1)-μ_2	sql	No-interpenetration	Not reported
**[MLL']**	HEWMOP	[Zn(bimbz)(glu)]. $2H_2O$[22]	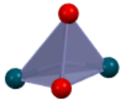 Mononulcear	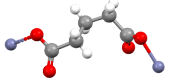 (κ^1)-(κ^1)-μ_2	sql	2-fold interpenetration	Not reported
**[MLL']**	IDUKOK	[Zn(bib)(glu)].4 H_2O[23]	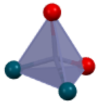 Mononulcear	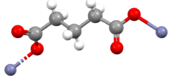 (κ^1)-(κ^1)-μ_2	sql	No-interpenetration	Not reported
**[MLL']**	GINTAA	[Zn(bix)(glu)].2 H_2O[24]	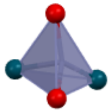 Mononulcear	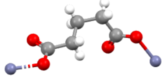 (κ^1)-(κ^1)-μ_2	sql	2-fold interpenetration	Not reported

**[MLL']**	FIMYIL	[Cd(bidpe)(glu)] . H_2O[25]	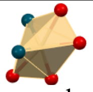 Mononulcear	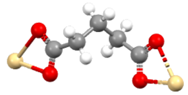 (κ^{2})-(κ^{2})-μ_{2}	sql	No-interpenetration	Not reported
**[MLL']**	IDUKIE	[Cd(bib)(glu)].4 H_2O[23]	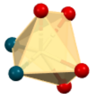 Mononulcear	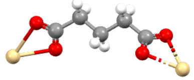 (κ^{2})-(κ^{2})-μ_{2}	sql	Non-interpenetrated	CO_2 and N_2 isotherm are reported
**[MLL']**	PEVKAD	[Co(bimb)(adi)] [26]	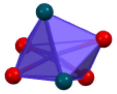 Mononulcear	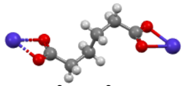 (κ^{2})-(κ^{2})-μ_{2}	dia	No-interpenetration	Not reported
**[MLL']**	RAXLUZ	[Cu(bib)(adi)].3 H_2O[27]	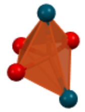 Mononulcear	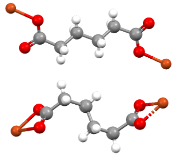 (κ^{1})-(κ^{1})-μ_{2} (κ^{2})-(κ^{2})-μ_{2}	sql	No-interpenetration	Not reported
**[MLL']**	CIWDIY	[Zn(bipmo)(adi)].2H_2O[28]	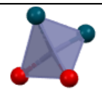 Mononulcear	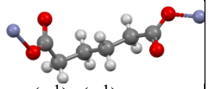 (κ^{1})-(κ^{1})-μ_{2}	cds	2-fold interpenetration	Not reported
**[MLL']**	JIQHEZ	[Zn(bipmo)(adi)].H_2O[28]	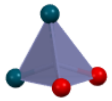 Mononulcear	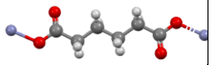 (κ^{1})-(κ^{1})-μ_{2}	sql	2-fold interpenetration	Not reported
**[MLL']**	RIDBOX	[Zn(bib)(adi)]. DMF.2H_2O[29]	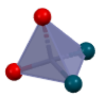 Mononulcear	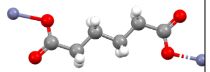 (κ^{1})-(κ^{1})-μ_{2}	hcb	6-fold interpenetration	Not reported
**[MLL']**	VAVKAG	[Zn(bime)(adi)]. 2.5H_2O[30]	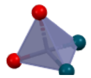 Mononulcear	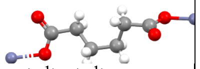 (κ^{1})-(κ^{1})-μ_{2}	sql	No-interpenetration	Not reported

**[MLL']**	KIHDOX	[Zn(bib)(adi)][31]	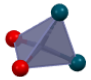 Mononulcear	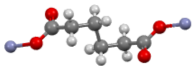 (κ^1)-(κ^1)-μ_2	dia	4-fold interpenetration	Not reported
**[MLL']**	PEVJUW	[Cd(bimb)(adi)][26]	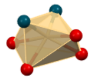 Mononulcear	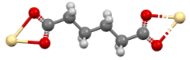 (κ^2)-(κ^2)-μ_2	dia	No-interpenetration	Not reported
**[MLL'(H_2O)]**	ACALAV	[Cd(adi)(NDI-A)(H_2O)][32]	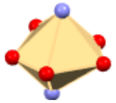 Mononulcear	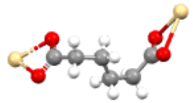 (κ^2)-(κ^2)-μ_2	sql	No-interpenetration	Not reported
**[MLL'(H_2O)]**	DAXZUY	[Cu(adi)(bipy)(H_2O)][33]	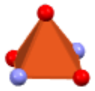 Mononulcear	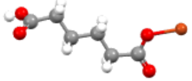 (κ^1)-μ_2	sql	2-fold interpenetration	Not reported
**[MLL'(H_2O)]**	OVECEY	[Cd(adipate)(H_2bpmp)(H_2O)]SO_4·$4H_2O$[12]	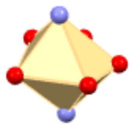 Mononulcear	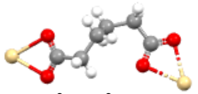 (κ^2)-(κ^2)-μ_2	sql	No-interpenetration	Not reported
**[MLL'(H_2O)]**	XUJRAX	[Zn(H_2O)(ADA)(1,4-bpetmb)][19]	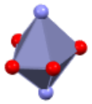 Mononulcear	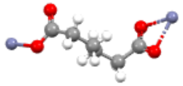 (κ^1)-(κ^2)-μ_2	sql	No-interpenetration	Not reported
**[MLL'(H_2O)]**	CEMFUY	[Cd(adi)(bpbde)(H_2O)][34]	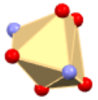 Mononulcear	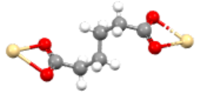 (κ^2)-(κ^2)-μ_2	dia	3-fold interpenetration	Not reported
**[MLL'(H_2O)]**	RUXLAZ	[Cd(adi)(bpp)(H_2O)][35]	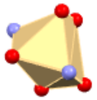 Mononulcear	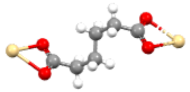 (κ^2)-(κ^2)-μ_2	zst	No-interpenetration	Not reported

**[MLL'(H_2O)]**	RUDQEP	[Cd(adi)(bpa)(H_2O)][36]	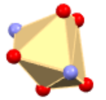 Mononulcear	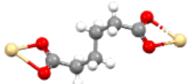 (κ^2)-(κ^2)-μ_2	zst	No-interpenetration	Not reported
**[MLL'(H_2O)]**	OVECIC	[Co(adi)(bpmp)(H_2O)][12]	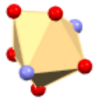 Mononulcear	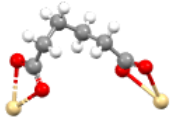 (κ^2)-(κ^2)-μ_2	$(4.6^2)(4.6^6 8^3)$	No-interpenetration	Not reported
**[MLL'(H_2O)]**	OVECUO	[Ni(adi)(bpmp)(H_2O)][12]	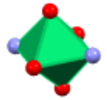 Mononulcear	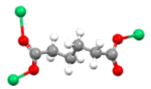 $(\kappa^1$- $\kappa^1)$-(κ^1)-μ_3	$(4.6^2)(4.6^6 8^3)$	No-interpenetration	Not reported
**[MLL'(H_2O)]**	EHOXAC	[Cd(muc)(azmebp)(H_2O)][37]	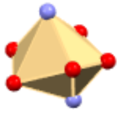 Mononulcear	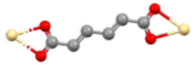 (κ^2)-(κ^2)-μ_2	sql	2-fold interpenetration	Not reported
**[MLL'($H_2O)_2$]**	AYOXOD	[Co(muc)(bpe)($H_2O)_2$][38]	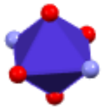 Mononulcear	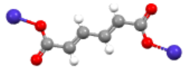 (κ^1)-(κ^1)-μ_2	sql	No-interpenetration	Not reported
**[MLL'($H_2O)_2$]**	NEHJIU	[Ni(muc)(bipy)($H_2O)_2$][39]	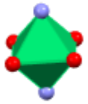 Mononulcear	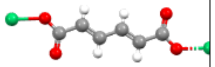 (κ^1)-(κ^1)-μ_2	sql	3-fold interpenetration	Not reported
**[MLL'($H_2O)_2$]**	ZARWAT	[Cd(muc)(bipy)($H_2O)_2$][40]	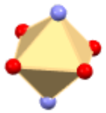 Mononulcear	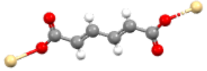 (κ^1)-(κ^1)-μ_2	sql	Non-interpenetrated	Not reported

**[MLL'(H_2O)$_2$]**	AYOXUJ	[Co(muc)(bpa)(H_2O)$_2$][41]	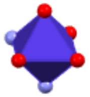 Mononulcear	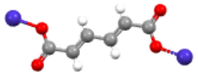 (κ^1)-(κ^1)-μ_2	neb	3-fold interpenetration	CO_2, N_2, Ar, H_2 adsorption was reported
**[MLL'(H_2O)$_2$]**	EMAMIQ	[Ni(muc)(bpa)(H_2O)$_2$][42]	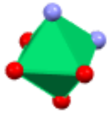 Mononulcear	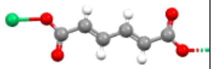 (κ^1)-(κ^1)-μ_2	neb	3-fold interpenetration	CO_2, N_2, Ar, H_2 adsorption was reported
**[MLL'(H_2O)$_2$]**	EMAMO W	[Ni(muc)(bpe)(H_2O)$_2$][42]	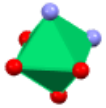 Mononulcear	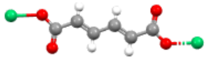 (κ^1)-(κ^1)-μ_2	neb	3-fold interpenetration	CO_2, N_2, Ar, H_2 adsorption was reported
**[MLL'(H_2O)$_2$]**	EMAMUC	[Ni(muc)(azobp) (H_2O)$_2$][42]	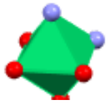 Mononulcear	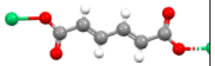 (κ^1)-(κ^1)-μ_2	neb	3-fold interpenetration	CO_2, N_2, Ar, H_2 adsorption was reported
**[MLL'(H_2O)$_2$]**	PAYXEW	[Cu(muc)(bpa)(H_2O)$_2$][43]	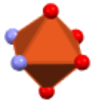 Mononulcear	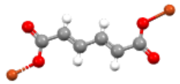 (κ^1)-(κ^1)-μ_2	neb	3-fold interpenetration	Not reported
**[MLL'(H_2O)$_2$]**	QOHTUD	[Co(muc)(bpe)(H_2O)$_2$][44]	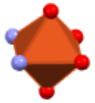 Mononulcear	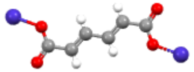 (κ^1)-(κ^1)-μ_2	neb	3-fold interpenetration	Not reported
**[MLL'(H_2O)$_2$]**	CIDBAU	[Cu(adi)(bipy)(H_2O)$_2$][45]	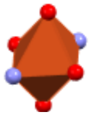 Mononulcear	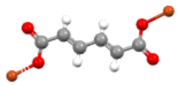 (κ^1)-(κ^1)-μ_2	cds	3-fold interpenetration	Not reported
**[MLL'(H_2O)$_2$]**	PILHUP	[Cu(bimbz)(adi)(H_2O)$_2$][46]	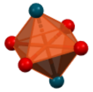 Mononulcear	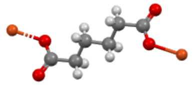 (κ^1)-(κ^1)-μ_2	sql	Non-interpenetrated	Not reported

**$[M_2L_2L']$**	MABJOP	$[Cu_2(Glu)_2(bipy)]$[47]	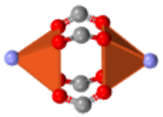 Paddlewheel Binulcear	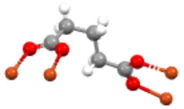 $(\kappa^1- \kappa^1)- (\kappa^1- \kappa^1)-\mu_4$	rob	No-interpenetration	Not reported
**$[M_2L_2L']$**	ACAJIZ	$[Cu_2(Glu)_2(bpa)]$[48]	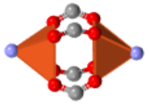 Paddlewheel Binulcear	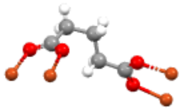 $(\kappa^1- \kappa^1)- (\kappa^1- \kappa^1)-\mu_4$	rob	No-interpenetration	CO_2, N_2, H_2 adsorption was reported
**$[M_2L_2L']$**	ACAJOF	$[Cu_2(Glu)_2(bpp)]$[48]	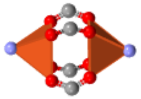 Paddlewheel Binulcear	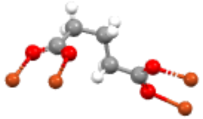 $(\kappa^1- \kappa^1)- (\kappa^1- \kappa^1)-\mu_4$	rob	No-interpenetration	CO_2, N_2, H_2 adsorption was reported
**$[M_2L_2L']$**	HAPJIV	$[Cu_2(Glu)_2(azobipy)]$[49]	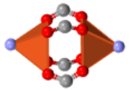 Paddlewheel Binulcear	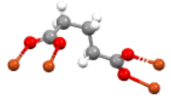 $(\kappa^1- \kappa^1)- (\kappa^1- \kappa^1)-\mu_4$	rob	No-interpenetration	Not reported
**$[M_2L_2L']$**	JOKFAT	$[Cu_2(Glu)_2(bpe)]$[50]	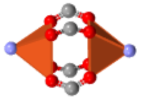 Paddlewheel Binulcear	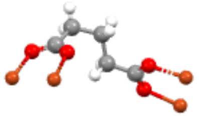 $(\kappa^1- \kappa^1)- (\kappa^1- \kappa^1)-\mu_4$	rob	No-interpenetration	Not reported
**$[M_2L_2L']$**	JOKJIF	$[Cu_2(Glu)_2(bipy)].2H_2O$[50]	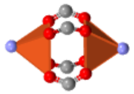 Paddlewheel Binulcear	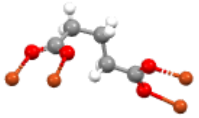 $(\kappa^1- \kappa^1)- (\kappa^1- \kappa^1)-\mu_4$	rob	No-interpenetration	Not reported

**[M_2L_2L']**	KEJKUJ	[$Cu_2(Glu)_2$(bpa)].$2H_2O$. NO[51]	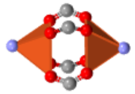 Paddlewheel Binulcear	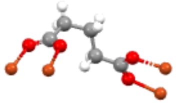 $(\kappa^1- \kappa^1)- (\kappa^1- \kappa^1)-\mu_4$	rob	No-interpenetration	Not reported
**[M_2L_2L']**	LEYTUI	[$Cu_2(Glu)_2$(4-pmi)].$4H_2O$[52]	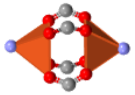 Paddlewheel Binulcear	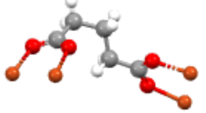 $(\kappa^1- \kappa^1)- (\kappa^1- \kappa^1)-\mu_4$	rob	No-interpenetration	CO_2, N_2, H_2, CH_4 adsorption was reported
**[M_2L_2L']**	LOXVUR	[$Cu_2(Glu)_2$(bipy)].$2CO_2$[53]	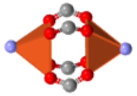 Paddlewheel Binulcear	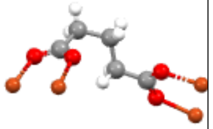 $(\kappa^1- \kappa^1)- (\kappa^1- \kappa^1)-\mu_4$	rob	No-interpenetration	CO_2 adsorption was reported
**[M_2L_2L']**	LOXWAY	[$Cu_2(Glu)_2$(bpe)].$2CO_2$[53]	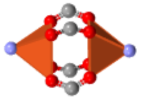 Paddlewheel Binulcear	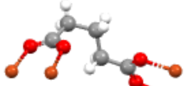 $(\kappa^1- \kappa^1)- (\kappa^1- \kappa^1)-\mu_4$	rob	No-interpenetration	CO_2 adsorption was reported
**[M_2L_2L']**	LOXWEC	[$Cu_2(Glu)_2$(azebipy)].$2CO_2$[53]	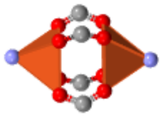 Paddlewheel Binulcear	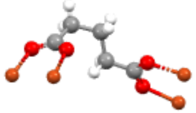 $(\kappa^1- \kappa^1)- (\kappa^1- \kappa^1)-\mu_4$	rob	No-interpenetration	CO_2 adsorption was reported
**[M_2L_2L']**	MABJUV	[$Cu_2(Glu)_2$(bipy)]. $3H_2O$[47]	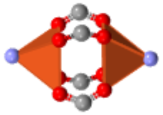 Paddlewheel Binulcear	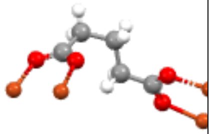 $(\kappa^1- \kappa^1)- (\kappa^1- \kappa^1)-\mu_4$	rob	No-interpenetration	Not reported

**[M_2L_2L']**	MABKEG	[$Cu_2(Glu)_2(bpe)$].$2H_2O$[47]	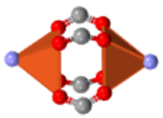 Paddlewheel Binulcear	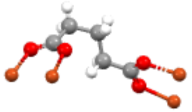 $(\kappa^1- \kappa^1)- (\kappa^1- \kappa^1)-\mu_4$	rob	No-interpenetration	Not reported
**[M_2L_2L']**	MABKIK	[$Cu_2(Glu)_2(bpe)$].$3H_2O$[47]	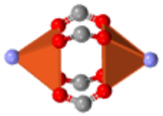 Paddlewheel Binulcear	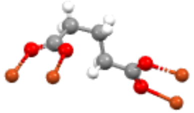 $(\kappa^1- \kappa^1)- (\kappa^1- \kappa^1)-\mu_4$	rob	No-interpenetration	Not reported
**[M_2L_2L']**	MIJBAK	[$Cu_2(Glu)_2(bpp)$][54]	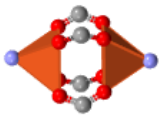 Paddlewheel Binulcear	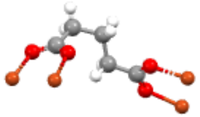 $(\kappa^1- \kappa^1)- (\kappa^1- \kappa^1)-\mu_4$	rob	No-interpenetration	CO_2, N_2, CH_4 adsorption was reported
**[M_2L_2L']**	NEHREA	[$Cu_2(Glu)_2(bpa)$][55]	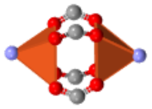 Paddlewheel Binulcear	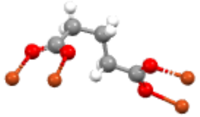 $(\kappa^1- \kappa^1)- (\kappa^1- \kappa^1)-\mu_4$	rob	No-interpenetration	CO_2, CH_4 adsorption was reported (Stepped isotherm for CO_2)
**[M_2L_2L']**	NEHRIE	[$Cu_2(Glu)_2(bpa)$][55]	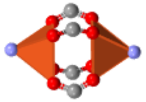 Paddlewheel Binulcear	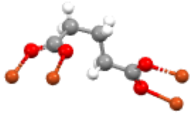 $(\kappa^1- \kappa^1)- (\kappa^1- \kappa^1)-\mu_4$	rob	No-interpenetration	CO_2, CH_4 adsorption was reported (Stepped isotherm for CO_2)
**[M_2L_2L']**	NEHROK	[$Cu_2(Glu)_2(bpa)$].CO_2.H_2O[55]	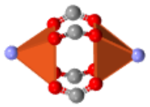 Paddlewheel Binulcear	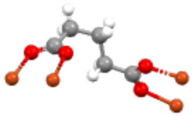 $(\kappa^1- \kappa^1)- (\kappa^1- \kappa^1)-\mu_4$	rob	No-interpenetration	CO_2, CH_4 adsorption was reported (Stepped isotherm for CO_2)

**[M_2L_2L']**	NEHRUQ	[$Cu_2(Glu)_2$(bpa)][55]	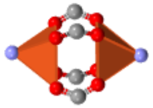 Paddlewheel Binulcear	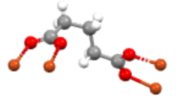 $(\kappa^1-\kappa^1)-(\kappa^1-\kappa^1)-\mu_4$	rob	No-interpenetration	CO_2, CH_4 adsorption was reported (Stepped isotherm for CO_2)
**[M_2L_2L']**	NEHSAX	[$Cu_2(Glu)_2$(bpp)]. (CH_3COCH_3)[55]	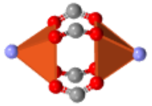 Paddlewheel Binulcear	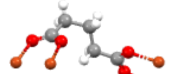 $(\kappa^1-\kappa^1)-(\kappa^1-\kappa^1)-\mu_4$	rob	No-interpenetration	CO_2, CH_4 adsorption was reported
**[M_2L_2L']**	NEHSEB	[$Cu_2(Glu)_2$(bpp)]. CO_2[55]	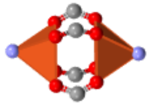 Paddlewheel Binulcear	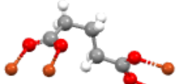 $(\kappa^1-\kappa^1)-(\kappa^1-\kappa^1)-\mu_4$	rob	No-interpenetration	CO_2, CH_4 adsorption was reported
**[M_2L_2L']**	NEHSIF	[$Cu_2(Glu)_2$(bpp)][55]	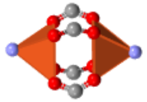 Paddlewheel Binulcear	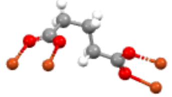 $(\kappa^1-\kappa^1)-(\kappa^1-\kappa^1)-\mu_4$	rob	No-interpenetration	CO_2, CH_4 adsorption was reported
**[M_2L_2L']**	OFECIO	[$Cu_2(Glu)_2$(azobipy)][56]	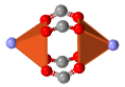 Paddlewheel Binulcear	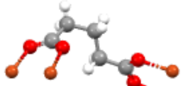 $(\kappa^1-\kappa^1)-(\kappa^1-\kappa^1)-\mu_4$	rob	No-interpenetration	CO_2, H_2, CH_4 adsorption was reported
**[M_2L_2L']**	OFEDAH	[$Cu_2(Glu)_2$(bpo)][56]	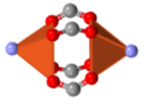 Paddlewheel Binulcear	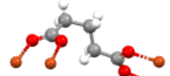 $(\kappa^1-\kappa^1)-(\kappa^1-\kappa^1)-\mu_4$	rob	No-interpenetration	CO_2, H_2, CH_4 adsorption was reported
**[M_2L_2L']**	RIPKIM	[$Cu_2(Glu)_2$(azmebipy)].H_2O[57]	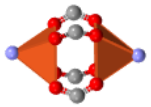	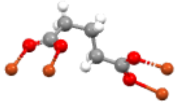	rob	No-interpenetration	CO_2, N_2, adsorption was reported

			Paddlewheel Binulcear	$(\kappa^1-\kappa^1)-(\kappa^1-\kappa^1)-\mu_4$			
**[M_2L_2L']**	RIPKOS	[$Cu_2(Glu)_2$(azmebipy)][57]	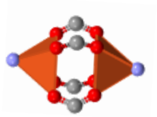 Paddlewheel Binulcear	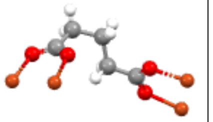 $(\kappa^1-\kappa^1)-(\kappa^1-\kappa^1)-\mu_4$	rob	No-interpenetration	CO_2, N_2, H_2O adsorption was reported
**[M_2L_2L']**	UZUNAF	[$Cu_2(Glu)_2$(azbipy)].$2H_2O$[58]	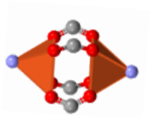 Paddlewheel Binulcear	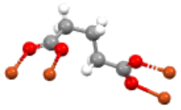 $(\kappa^1-\kappa^1)-(\kappa^1-\kappa^1)-\mu_4$	rob	No-interpenetration	CO_2, H_2O adsorption was reported
**[M_2L_2L']**	UZUNEJ	[$Cu_2(Glu)_2$(azbipy)][58]	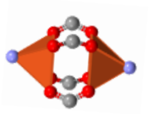 Paddlewheel Binulcear	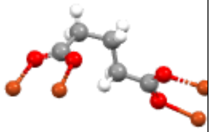 $(\kappa^1-\kappa^1)-(\kappa^1-\kappa^1)-\mu_4$	rob	No-interpenetration	CO_2, H_2O adsorption was reported
**[M_2L_2L']**	WOCWAN	[$Cu_2(Glu)_2$(bpe)][59]	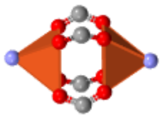 Paddlewheel Binulcear	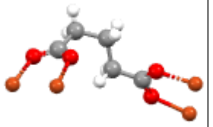 $(\kappa^1-\kappa^1)-(\kappa^1-\kappa^1)-\mu_4$	rob	No-interpenetration	H_2O and MeOH adsorption were reported
**[M_2L_2L']**	WOHBOL	[$Cu_2(Glu)_2$(bpe)][59]	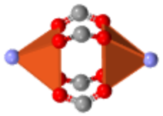 Paddlewheel Binulcear	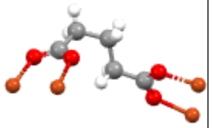 $(\kappa^1-\kappa^1)-(\kappa^1-\kappa^1)-\mu_4$	rob	No-interpenetration	H_2O and MeOH adsorption were reported

**[M_2L_2L']**	INALOA	[$Cu_2(adi)_2(bpa)$][60]	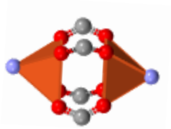 Paddlewheel Binulcear	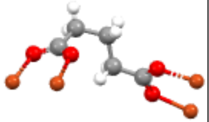 $(\kappa^1- \kappa^1)- (\kappa^1- \kappa^1)-\mu_4$	rob	No-interpenetration	Not reported
**[M_2L_2L']**	FIGQUJ	[$Cu_2(glu)_2$(3-pina)]· $3.5H_2O$[61]	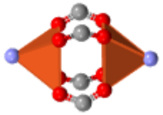 Paddlewheel Binulcear	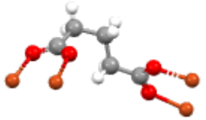 $(\kappa^1- \kappa^1)- (\kappa^1- \kappa^1)-\mu_4$	pcu	No-interpenetration	Not reported
**[M_2L_2L']**	GEHQIU	[$Zn_2(Glu)_2$(bpp)][62]	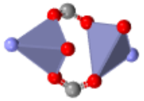 Binulcear	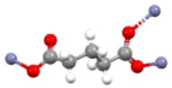 $(\kappa^1)- (\kappa^1- \kappa^1)-\mu_3$	pcu	2-fold interpenetration	Not reported
**[M_2L_2L']**	TOTTUU	[$Co_4(glu)_4(bpp)_2$][63]	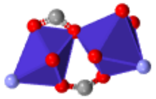 Corner shared binulcear	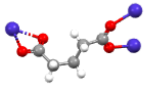 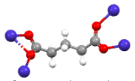 $(\kappa^2)- (\kappa^1- \kappa^1)-\mu_3$ $(\kappa^1- \kappa^2)- (\kappa^1- \kappa^1)-\mu_4$	pcu	2-fold interpenetration	Not reported
**[M_2L_2L']**	OQUGIT	[Co(bix)(glu)][20]	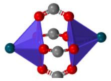 Paddlewheel Binulcear	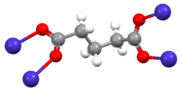 $(\kappa^1- \kappa^1)- (\kappa^1- \kappa^1)-\mu_4$	pcu	No-interpenetration	Not reported
**[M_2L_2L']**	KIFRUO	[Cu(4-bpfp)0.5(glu)]· H_2O[64]	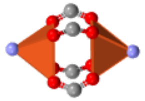 Paddlewheel Binulcear	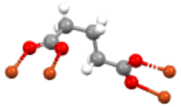 $(\kappa^1- \kappa^1)- (\kappa^1- \kappa^1)-\mu_4$	mab	No-interpenetration	Not reported

**[M_2L_2L']**	HOJLEY	[$Cu_2(glu)_2(bpmp)]\cdot 4H_2O$[65]	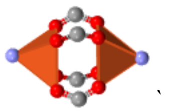 Paddlewheel Binulcear	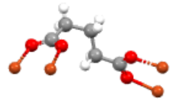 (κ^1- κ^1)- (κ^1- κ^1)-μ_4	mab	No-interpenetration	Not reported
**[$M_2L_2L'_2$]**	AYALUH	[Mn(Glu)(bipy)][66]	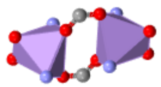 Binulcear	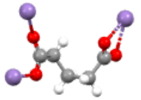 (κ^2)- (κ^1- κ^1)-μ_3	sql	No-interpenetration	Not reported
**[$M_2L_2L'_2$]**	ECINEM	[Cu(glu)(bpb)][67]	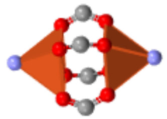 Paddlewheel Binulcear	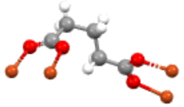 (κ^1- κ^1)- (κ^1- κ^1)-μ_4	sql	No-interpenetration	Not reported
**[$M_2L_2L'_2$]**	GINWOQ	[Cd(glu)(bipy)][68]	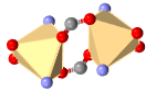 Binulcear	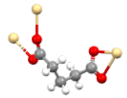 (κ^2)- (κ^1- κ^1)-μ_3	sql	No-interpenetration	Not reported
**[$M_2L_2L'_2$]**	KALMUG	[Cu(glu)(bipy)][69]	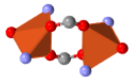 Binulcear	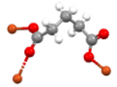 (κ^1- κ^1)- (κ^1)-μ_3	sql	No-interpenetration	Not reported
**[$M_2L_2L'_2$]**	KALNAN	[Zn(Glu)(Bipy)][69]	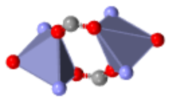 Binulcear	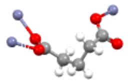 (κ^1- κ^1)- (κ^1- κ^1)-μ_4	sql	No-interpenetration	Not reported
**[$M_2L_2L'_2$]**	NIYPAO	[Cd(Glu)(azpy)][70]	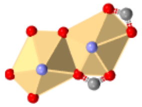 Edge shared binulcear	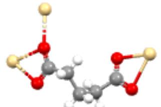 (κ^1- κ^2)- (κ^1- κ^1)-μ_4	sql	No-interpenetration	CO_2, H_2 adsorption was reported

**[$M_2L_2L'_2$]**	NUYBAL	[Cd(Glu)(bpt)][71]	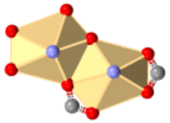 Edge shared binulcear	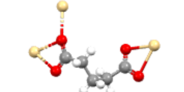 (κ^1- κ^1)- (κ^1- κ^1)-μ_4	sql	No-interpenetration	Not reported
**[$M_2L_2L'_2$]**	QUTTIJ	[Co(Glu)(bpt)][72]	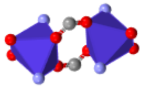 Binulcear	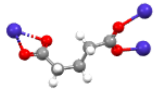 (κ^2)- (κ^1- κ^1)-μ_3	sql	No-interpenetration	Not reported
**[$M_2L_2L'_2$]**	TOTVAC	[$Co_2(Glu)_2$(μ-bpe)$_2$]·$(H_2O)_{0.5}$[73]	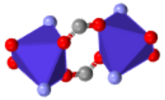 Binulcear	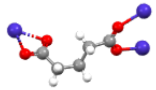 (κ^2)- (κ^1- κ^1)-μ_3	sql	No-interpenetration	Not reported
**[$M_2L_2L'_2$]**	TOTTOO	[$Co_2(Glu)_2$(μ-bpa)$_2$]·$(H_2O)_4$[73]	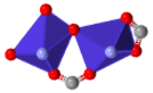 Corner shared binulcear	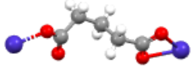 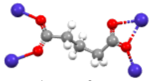 (κ^1)-(κ^2)-μ_2 (κ^1- κ^1)-(κ^1- κ^1)-μ_4	pcu	2-fold interpenetration	Not reported
**[$M_2L_2L'_2$]**	YOFRER	[Cd(glutarate)(dpa)][74]	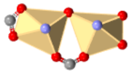 Corner shared binulcear	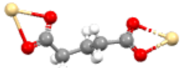 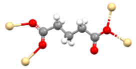 (κ^2)-(κ^2)-μ_2 (κ^1- κ^1)-(κ^1- κ^1)-μ_4	pcu	2-fold interpenetration	Not reported
**[$M_2L_2L'_2$]**	IBOVEE	[Cud(adi)(mpnd))][75]	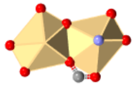 Edge shared binulcear	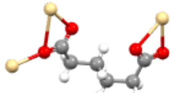 (κ^2)- (κ^1- κ^2)-μ_3	sql	No-interpenetration	Not reported

**$[M_2L_2L'_2]$**	NOWYUW	[Cd(adi)(bpbde)][76]	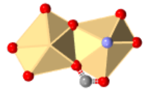 Edge shared binulcear	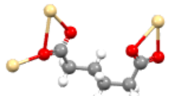 (κ^2)- $(\kappa^1$- $\kappa^2)$-μ_3	sql	No-interpenetration	Not reported
**$[M_2L_2L'_2]$**	NOWZAD	[Cu(adi)(tptco)][76]	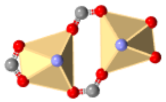 Binulcear	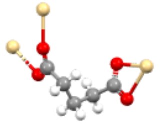 (κ^2)- $(\kappa^1$- $\kappa^1)$-μ_3	sql	No-interpenetration	Not reported
**$[M_2L_2L'_2]$**	WELMIN	[Zn(4-pmi)(adp)]·$(H_2O)_{0.5}$[77]	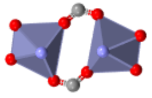 Binulcear	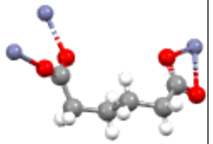 (κ^2)- $(\kappa^1$- $\kappa^1)$-μ_3	sql	No-interpenetration	Not reported
**$[M_2L_2L'_2]$**	XIDRAF	[Cd(4-pmi)(adp)]·$(H_2O)_{0.5}$[78]	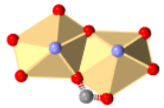 Edge shared binulcear	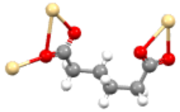 (κ^2)- $(\kappa^1$- $\kappa^2)$-μ_3	sql	No-interpenetration	Not reported
**$[M_2L_2L'_2]$**	AYAMAO	[Mn(adi)(bipy)][79]	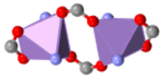 Binulcear	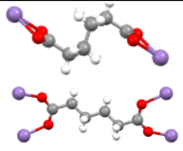 (κ^2)-(κ^2)-μ_2 $(\kappa^1$- $\kappa^1)$-$(\kappa^1$- $\kappa^1)$-μ_4	pcu	2-fold interpenetrated	Not reported
**$[M_2L_2L'_2]$**	BETDUA	[Co(adi)(bipy)][80]	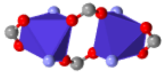 Binulcear	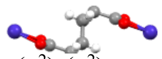 (κ^2)-(κ^2)-μ_2	pcu	2-fold interpenetrated	Not reported

**[$M_2L_2L'_2$]**	EBOBOP	[Zn(adi)(bpe)][81]	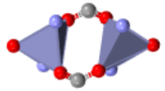 Binulcear	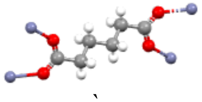 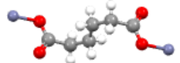 (κ^1- κ^1)-(κ^1- κ^1)-μ_4 (κ^1)-(κ^1)-μ_2	pcu	2-fold interpenetrated	CO_2, H_2, N_2 adsorption was reported
**[$M_2L_2L'_2$]**	GINWUW	[Cd(adi)(bipy)][82]	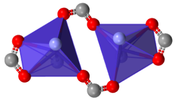 Binulcear	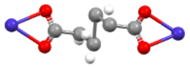 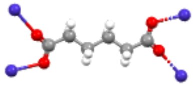 (κ^2)-(κ^2)-μ_2 (κ^1- κ^1)-(κ^1- κ^1)-μ_4	pcu	2-fold interpenetrated	Not reported
**[$M_2L_2L'_2$]**	KALNER	[Zn(adi)(bipy)][83]	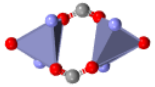 Binulcear	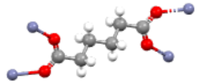 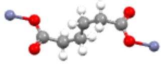 (κ^1- κ^1)-(κ^1- κ^1)-μ_4 (κ^1)-(κ^1)-μ_2	pcu	2-fold interpenetrated	Not reported
**[$M_2L_2L'_2$]**	LAYLIH	[Cu(adi)(bipy)][84]	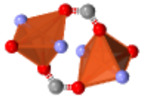 Binulcear	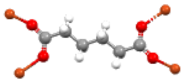 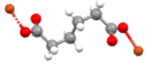 (κ^1- κ^1)-(κ^1- κ^1)-μ_4 (κ^1)-(κ^1)-μ_2	pcu	2-fold interpenetrated	Not reported
**[$M_2L_2L'_2$]**	LECBOL	[Cu(adi)(bipy)][84]	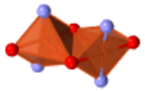	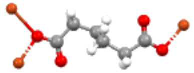	pcu	2-fold interpenetrated	Not reported

			Edge shared binulcear	(κ^2)-(κ^1)-μ_3			
**[$M_2L_2L'_2$]**	LIPJIE	[Co(adi)(bipy)][14]	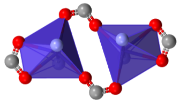 Binulcear	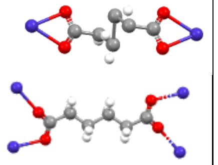 (κ^2)-(κ^2)-μ_2 $(\kappa^1$- $\kappa^1)$-(κ^1- $\kappa^1)$-μ_4	pcu	2-fold interpenetrated	Not reported
**[$M_2L_2L'_2$]**	PECJIS	[Cd(adp)(bpfp)][85]	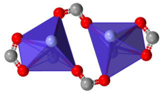 Binulcear	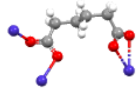	pcu	Non-interpenetrated	Not reported
**[$M_2L_2L'_2$]**	RIYBIL	[Ni(adi)(bipy)][86]	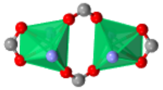 Binulcear	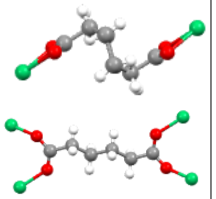 (κ^2)-(κ^2)-μ_2 $(\kappa^1$- $\kappa^1)$-(κ^1- $\kappa^1)$-μ_4	pcu	2-fold interpenetrated	Not reported
**[$M_2L_2L'_2$]**	TABQIX	[Mn(adi)(bpa)][87]	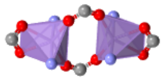 Binulcear	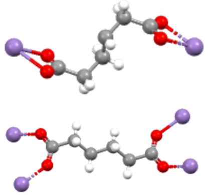 (κ^2)-(κ^2)-μ_2 $(\kappa^1$- $\kappa^1)$-(κ^1- $\kappa^1)$-μ_4	pcu	2-fold interpenetrated	Not reported

**$[M_2L_2L'_2]$**	YUNPAZ	[Co(adi)(dpa)][88]	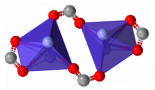<br>Binulcear	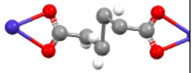<br>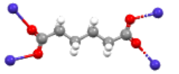<br>(κ^2)-(κ^2)-μ_2<br>$(\kappa^1$- $\kappa^1)$-(κ^1- $\kappa^1)$-<br>μ_4	pcu	2-fold interpenetrated	Not reported
**$[M_2L_2L'_2]$**	AYOYIY	[Co(muc)(azbpy)][89]	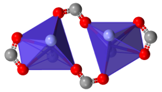<br>Binulcear	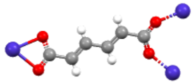<br>(κ^2)- $(\kappa^1$- $\kappa^1)$-μ_3	pcu	2-fold interpenetrated	Not reported
**$[M_2L_2L'_2]$**	AZILEC	[Co(muc)(azobipy)][90]	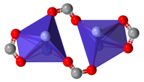<br>Binulcear	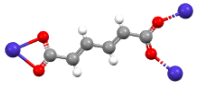<br>(κ^2)- $(\kappa^1$- $\kappa^1)$-μ_3	pcu	2-fold interpenetrated	CO_2, H_2, N_2 , Ar adsorption was reported
**$[M_2L_2L'_2]$**	PAYXIA	[Cu(muc)(azbpy)][43]	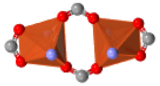<br>Binulcear	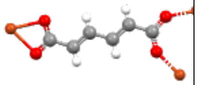<br>(κ^2)- $(\kappa^1$- $\kappa^1)$-μ_3	pcu	2-fold interpenetrated	Not reported
**$[M_2L_2L'_2]$**	QIVZEC	[Zn(muc)(azmebpy)][15]	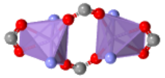<br>Binulcear	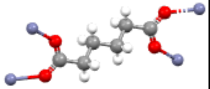<br>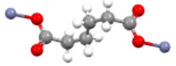<br>(κ^1)-(κ^1)-μ_2<br>$(\kappa^1$- $\kappa^1)$-(κ^1- $\kappa^1)$-<br>μ_4	pcu	2-fold interpenetrated	Not reported
**$[M_2L_2L'_2]$**	QOHTOX	[Co(muc)(bpe)][44]	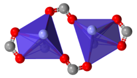<br>Binulcear	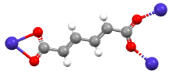<br>(κ^2)- $(\kappa^1$- $\kappa^1)$-μ_3	pcu	2-fold interpenetrated	Not reported
**$[M_2L_2L'_2]$**	SAKCIT	[Co(muc)(azmebpy)][91]	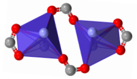	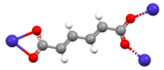<br>(κ^2)- $(\kappa^1$- $\kappa^1)$-μ_3	pcu	2-fold interpenetrated	Not reported

			Binulcear				
**$[M_2L_2L'_2]$**	SUJQOE	[Zn(muc)(bpe)][92]	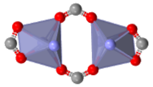 Binulcear	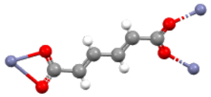 (κ^2)- $(\kappa^1$- $\kappa^1)$-μ_3	pcu	2-fold interpenetrated	Not reported
**$[M_2L_2L'_2]$**	SUJREV	[Zn(muc)(rctt-tpcb)][92]	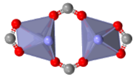 Binulcear	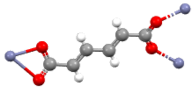 (κ^2)- $(\kappa^1$- $\kappa^1)$-μ_3	pcu	2-fold interpenetrated	Not reported
**$[M_2L_2L'_2]$**	YONRIE	[Cd(imb)(glu)]. H_2O[93]	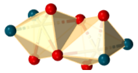 ` Edge shared binulcear	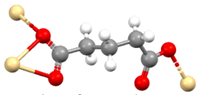 $(\kappa^1$- $\kappa^2)$- (κ^1)-μ_3	sql	Non-interpenetrated	Not reported
**$[M_2L_2L'_2]$**	JAWJOK	[Cd(1,3-bipz)(adi)][94]	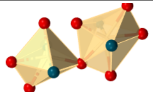 Corner shared binulcear	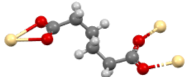 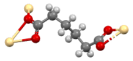 (κ^2)- $(\kappa^1$- $\kappa^1)$-μ_3 $(\kappa^1$- $\kappa^2)$- (κ^2)-μ_3	pcu	2-fold interpenetrated	Not reported

* Point symbols have been reported in the absence of a three-letter code for topologies.

Method (Ligand Synthesis)

The linker 1,4-bis-(1H-imidazol-1-yl)benzene (bimbz) was synthesized with modification of reported procedures.[95]

Synthesis of **bimbz**: 1,4-dibromobenzene (5.0 g, 21.2 mmol, 1.0 eq), CuI (805 mg, 20 mol%), imidazole (4.33 g, 63.6 mmol, 3.0 eq) and K_2CO_3 (8.78 g, 63.6 mmol, 3.0 eq) were all added to anhydrous DMF (50 ml) under N_2. The resulting reaction mixture was then heated to 150 °C for 48 h under an atmosphere of N_2. After cooling to room temperature, the mixture was diluted with DCM (250 ml) and filtered. The filtered organic layer was transferred to a large separating funnel and washed twice with H_2O (2 × 500 ml). After drying over $MgSO_4$, the organic layer was concentrated under reduced pressure. Final purification was achieved by rapid trituration of the compound from a DCM/hexane mixture, affording bimbz as a white solid.

Table S2 Crystal data and structure refinement details for solid **sql-glu-Ni**, **sql-adi-Ni** and **dia-muc-Ni**.

Parameter	**sql-glu-Ni**	**sql-adi-Ni**	**dia-muc-Ni**
Formula	C17 H16 N4 Ni O4.17	C18 H22 N4 Ni O6	C19 H18 N3 Ni O6
Formula weight	401.68	449.09	433.05
T (K)	102 K	100 K	101 K
Crystal system	Triclinic	Triclinic	Triclinic
Space group	$P\bar{1}$	$P\bar{1}$	$P\bar{1}$
Space group number	2	2	2
a (Å)	7.7152(2)	5.2255(2)	9.2250(2)
b (Å)	7.9573(1)	9.0139(3)	9.5660(2)
c (Å)	14.2571(3)	11.0579(3)	11.7477(3)
α (°)	91.217(1)	108.563(1)	70.798(1)
β (°)	104.774(1)	94.700(1)	79.692(1)
γ (°)	97.600(1)	104.557(1)	75.831(1)
V (Å^3)	837.52(3)	470.40(3)	943.73(4)
Z	2	1	2
Dcalc (gcm^{-3})	1.593	1.585	1.559
$\mu_{MoK\alpha}$ (cm^{-1})	1.191	1.077	1.071
Theta range (°)	2.76-28.37	2.90-28.31	2.90-28.31
R_1,[a] wR_2 [I > 2σ(I)][b]	0.0281, 0.0648	0.0189, 0.0433	0.0336, 0.0861
GOF	1.041	1.058	1.058
CCDC no.	2449933	2449934	2449935
[a]$R_1 = \sum\|\|F_o\| - \|F_c\|\|/\sum\|F_o\|$; [b]$wR_2 = \sum[w(F_o^2 - F_c^2)^2]/\sum[w(F_o^2)^2]^{1/2}$			

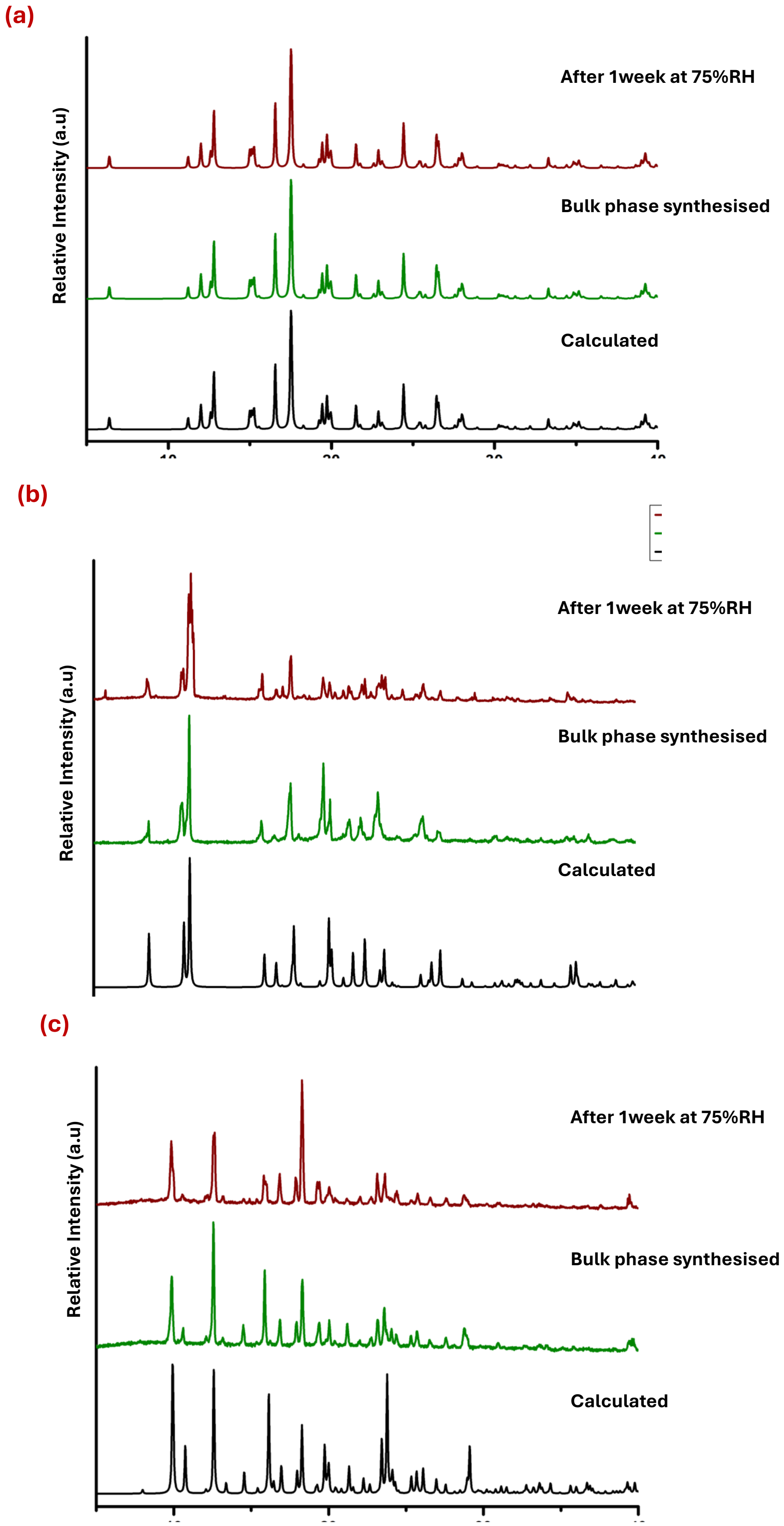
(a)
Relative Intensity (a.u)
After 1week at 75%RH
Bulk phase synthesised
Calculated
(b)
Relative Intensity (a.u)
After 1week at 75%RH
Bulk phase synthesised
Calculated
(c)
Relative Intensity (a.u)
After 1week at 75%RH
Bulk phase synthesised
Calculated

Figure S1 PXRD plots for (a) **sql-glu-Ni,** (b) **sql-adi-Ni** and (c) **dia-muc-Ni**, indicating the homogeneity of the bulk samples and effect on the phase purity when expose to 75% RH for one week.

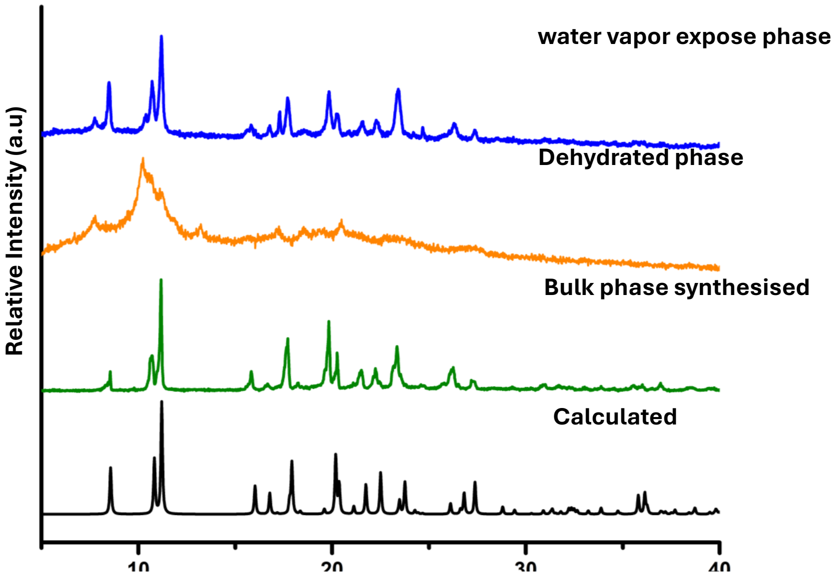

Figure S2 PXRD plots for **sql-adi-Ni** solid.

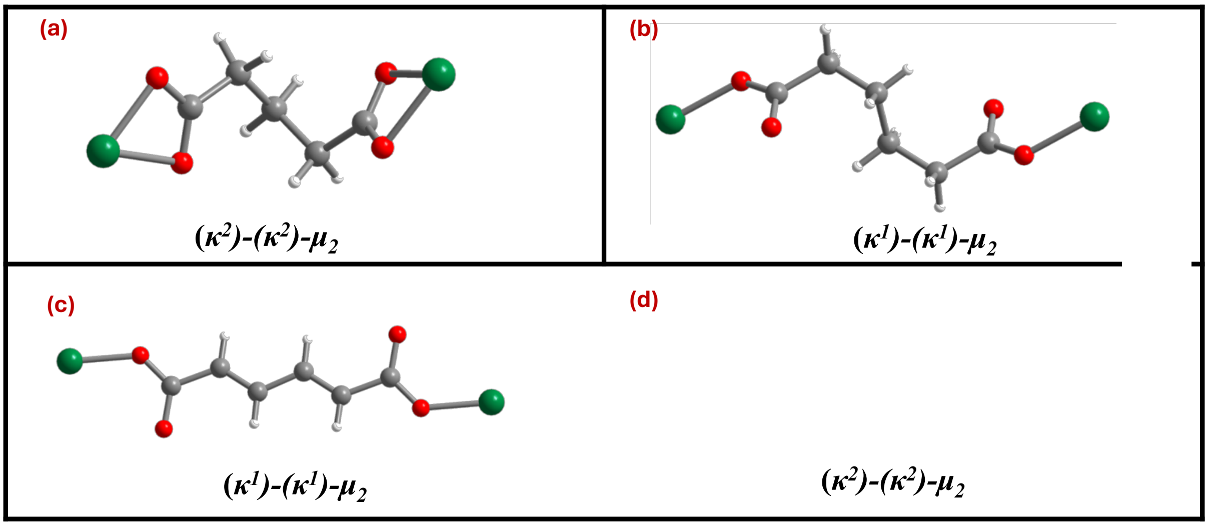

Scheme S2 Coordination modes of aliphatic acids (glu, adi and muc) in solids **sql-glu-Ni** (a), **sql-adi-Ni** (b) and **dia-muc-Ni** (c and d).

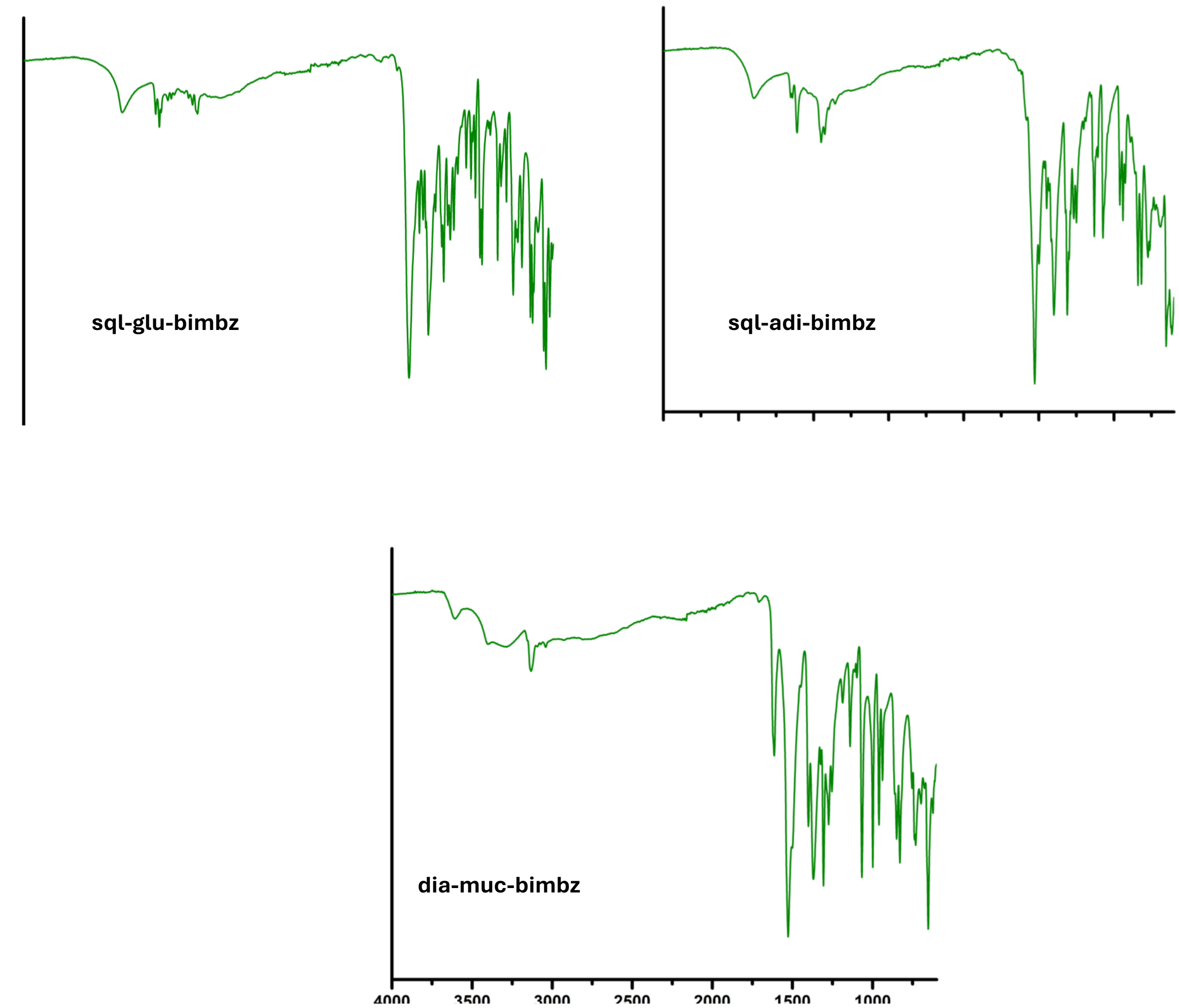

Figure S3 FTIR spectra for **sql-glu-Ni**, **sql-adi-Ni** and **dia-muc-Ni**

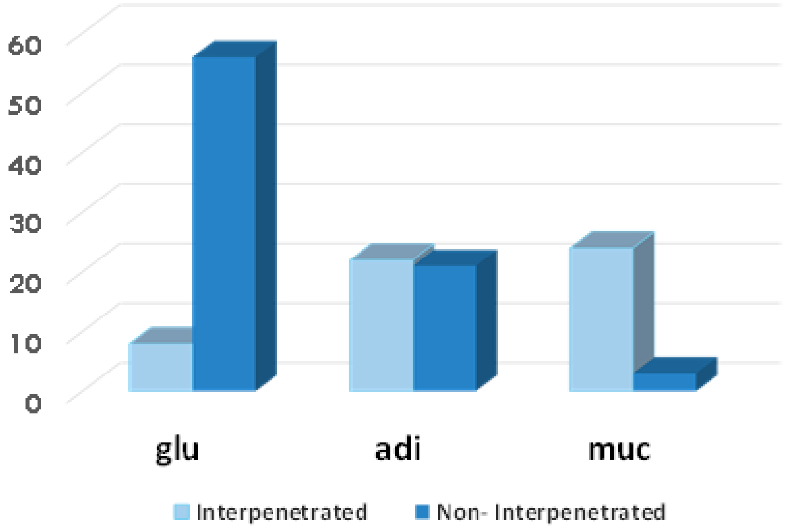

Figure S4 Histogram representing the number of interpenetration and non-interpenetration structure with **glu, adi** and **muc**.

Table S3: Selected bond lengths (Å) and angles (°) for solids.

**sql-glu-Ni**			
Ni1—O1	2.207(2)	O4^{i}—Ni1—O1	86.64(5)
Ni1—O4^{i}	2.141(2)	O2—Ni1—O1	61.86(5)
Ni1—O2	2.056(2)	O2—Ni1—O4^{i}	100.49(5)
Ni1—O5^{i}	2.084(2)	O2—Ni1—O5^{i}	161.24(6)
Ni1—N1	2.037(2)	O5^{i}—Ni1—O1	107.06(5)
Ni1—N3	2.050(2)	O5^{i}—Ni1—O4^{i}	62.41(5)
**sql-adi-Ni**			
Ni1—O1	2.081(1)	O1—Ni1—O3	91.51(4)
Ni1—O3	2.090(2)	N1—Ni1—O1	90.25(4)
Ni1—N1	2.064(2)	N1—Ni1—O3	91.91(5)
**dia-muc-Ni**			
Ni—O4	2.021(2)	O4—Ni—O3	101.88(6)
Ni—O3	2.128(2)	O4—Ni—O5	164.08(6)
Ni—O5	2.137(2)	O4—Ni—O1	92.38(5)
Ni—O1	2.120(2)	O4—Ni—N1	90.02(6)
Ni—N1	2.062(2)	O4—Ni—N3	94.89(6)
Ni—N3	2.032(2)		

Reference

1 Z.-R. Pan, Z.-Z. Shi, X.-J. Gao and H.-G. Zheng, *Inorg Chem Commun*, 2017, 86, 290–294.

2 D. Chen, Y. Zhong, C. Zhang, D. Xu and Z. Lin, *Inorg Chem Commun*, 2018, 92, 74–77.

3 I. H. Hwang, J. M. Bae, Y.-K. Hwang, H.-Y. Kim, C. Kim, S. Huh, S.-J. Kim and Y. Kim, *Dalton Trans.*, 2013, 42, 15645–15649.

4 J.-S. Hu, Z. Lei, H.-L. Xing, X.-M. Zhang, J.-J. Shi and J. He, *Mendeleev Communications*, 2013, 23, 229–230.

5 L. Zhu, F. Y. Yao and Y. Q. Zheng, *Koord.Kim*, 2012, 38, 473.

6 E. M. Low and R. L. LaDuca, *Inorganica Chim Acta*, 2015, 425, 221–232.

7 M. R. Montney, R. M. Supkowski, R. J. Staples and R. L. LaDuca, *J Solid State Chem*, 2009, 182, 8–17.

8 J.-S. Guo, G. Xu, S.-H. Wang, M.-S. Wang, M.-J. Zhang, G.-C. Guo and J.-S. Huang, *Inorg Chem Commun*, 2014, 45, 108–111.

9 A. L. Pochodylo, J. A. Wilson, J. W. Uebler, S. H. Qiblawi and R. L. LaDuca, *Inorganica Chim Acta*, 2014, 423, 298–306.

10 L.-S. Long, Y.-R. Wu, R.-B. Huang and L.-S. Zheng, *Inorg Chem*, 2004, 43, 3798–3800.

11 G.-L. Wen, Y.-Y. Wang, W.-H. Zhang, C. Ren, R.-T. Liu and Q.-Z. Shi, *CrystEngComm*, 2010, 12, 1238–1251.

12 A. Banisafar, D. P. Martin, J. S. Lucas and R. L. LaDuca, *Cryst Growth Des*, 2011, 11, 1651–1661.

13 I. H. Hwang, H.-Y. Kim, M. M. Lee, Y. J. Na, J. H. Kim, H.-C. Kim, C. Kim, S. Huh, Y. Kim and S.-J. Kim, *Cryst Growth Des*, 2013, 13, 4815–4823.

14 M. R. Montney, S. Mallika Krishnan, R. M. Supkowski and R. L. LaDuca, *Inorg Chem*, 2007, 46, 7362–7370.

15 C. M. Nagaraja, B. Ugale and A. Chanthapally, *CrystEngComm*, 2014, 16, 4805–4815.

16 B. Ugale and C. M. Nagaraja, *J Inorg Organomet Polym Mater*, 2014, 24, 1032–1037.

17 H.-C. Kim, S. Huh, J. Y. Kim, H. R. Moon, D. N. Lee and Y. Kim, *CrystEngComm*, 2017, 19, 99–109.

18 C. M. Nagaraja, B. Ugale and A. Chanthapally, *CrystEngComm*, 2014, 16, 4805–4815.

19 Y. Ge, W. Ma, N.-Y. Li, S.-J. Wang and D. Liu, *Inorganica Chim Acta*, 2015, 432, 32–40.

20 Y. Yu-Ting, T. Chang-Zheng, W. Fan, Z. Yue, W. Jian-Ling, Y. Hong-Ju and C. Fei-Xiang, *CHINESE JOURNAL OF INORGANIC CHEMISTRY*, 2021, 37, 735–743.

21 H.-J. Hao, D. Sun, F.-J. Liu, R.-B. Huang and L.-S. Zheng, *Cryst Growth Des*, 2011, 11, 5475–5482.

22 W. Si-Lu, L. Qian, Z. Cheng, W. Xiao, L. Si-Yu, H. Xiang-Yang, T. Long, Y. Er-Lin, F. Feng and W. Ji-Jiang, *J Solid State Chem*, 2022, 316, 123492.

23 F. Haque, A. Halder and D. Ghoshal, *Cryst Growth Des*, 2018, 18, 5231–5244.

24 G. X. Liu and Z. Q. Liu, *Russian Journal of Coordination Chemistry*, 2013, 39, 399–404.

25 J.-S. Hu, X. Zhuo, X.-H. Liu, H.-L. Xing, J. He and H.-G. Zheng, *Inorg Chem Commun*, 2013, 33, 15–18.

26 X.-Z. Sun, M.-H. Zeng, B. Wang, B.-H. Ye and X.-M. Chen, *J Mol Struct*, 2007, 828, 10–14.

27 S. Jin, W. Daqi and Y. and Xu, *J Coord Chem*, 65, 1953–1969.

28 G. F. Wang, *Russian Journal of Coordination Chemistry*, 2018, 44, 540–546.

29 D. Sun, M.-Z. Xu, S.-S. Liu, S. Yuan, H.-F. Lu, S.-Y. Feng and D.-F. Sun, *Dalton Trans.*, 2013, 42, 12324–12333.

30 H.-J. Hao, D. Sun, F.-J. Liu, R.-B. Huang and L.-S. Zheng, *J Mol Struct*, 2012, 1012, 131–136.

31 L. Chang-Qing, Y. Jin-Xia, Z. Xin, Q. Ye-Yan and Y. Yuan-Gen, *CHINESE JOURNAL OF STRUCTURAL CHEMISTRY*, 2018, 37, 1470–1478.

32 C. Wang, G. Zeng, Z.-X. You, Y.-H. Xing, F.-Y. Bai and L.-X. Sun, *Inorg Chem*, 2022, 61, 10066–10078.

33 J.-L. Lin and Y.-Q. Zheng, *Acta Crystallographica Section C*, 2005, 61, m501–m503.

34 Y.-X. Hou and D. Liu, *Acta Crystallographica Section C*, 2017, 73, 869–873.

35 J.-X. Yang, J.-Q. Zhai, X. Zhang, Y.-Y. Qin and Y.-G. Yao, *Dalton Trans.*, 2016, 45, 711–723.

36 N. Goel and N. Kumar, *Inorganica Chim Acta*, 2020, 503, 119352.

37 B. Ugale and C. M. Nagaraja, *RSC Adv.*, 2016, 6, 28854–28864.

38 B. Ugale and C. M. Nagaraja, CCDC 1511815,*CSD Communication*, 2016.

39 B. Chen, F. Jiang, L. Han, B. Wu, D. Yuan, M. Wu and M. Hong, *Inorg Chem Commun*, 2006, 9, 371–374.

40 B. Ugale and C. M. Nagaraja, CCDC 1415010, *CSD Communication*, 2016.

41 B. Ugale and C. M. Nagaraja, CCDC 1511816, *CSD Communication*, 2016.

42 B. Ugale, S. S. Dhankhar and C. M. Nagaraja, *Inorg Chem*, 2016, 55, 9757–9766.

43 D. Fu, S. Yang, J. Lu, H. Lian and K. Qin, *J Clust Sci*, 2022, 33, 529–536.

44 M. H. Mir, S. Kitagawa and J. J. Vittal, *Inorg Chem*, 2008, 47, 7728–7733.

45 M. H. Mir and J. J. Vittal, *Inorganica Chim Acta*, 2013, 403, 97–101.

46 C. Zhang, M. Wang, L. Liu, X. Yang and X. Xu, *Electrochem commun*, 2013, 33, 131–134.

47 B. Rather and M. J. Zaworotko, *Chem. Commun.*, 2003, 830–831.

48 I. H. Hwang, J. M. Bae, W.-S. Kim, Y. D. Jo, C. Kim, Y. Kim, S.-J. Kim and S. Huh, *Dalton Trans.*, 2012, 41, 12759–12765.

49 S. Yang, M. Veerana, N. Yu, W. Ketya, G. Park, S. Kim and Y. Kim, *Applied Sciences*, DOI:10.3390/app12010260.

50 J. H. Jo, H.-C. Kim, S. Huh, Y. Kim and D. N. Lee, *Dalton Trans.*, 2019, 48, 8084–8093.

51 D. N. Lee, Y. R. Kim, Y. Kim, B. J. Park, S. J. Lee, S.-J. Kim and J. H. Shin, *ACS Appl Bio Mater*, 2022, 5, 4301–4309.

52 A. Maiti, S. Dinda, A. Halder, P. Das and D. Ghoshal, *Cryst Growth Des*, 2023, 23, 1860–1867.

53 C. X. Bezuidenhout, V. J. Smith, P. M. Bhatt, C. Esterhuysen and L. J. Barbour, *Angewandte Chemie International Edition*, 2015, 54, 2079–2083.

54 J. M. Seco, D. Fairen-Jimenez, A. J. Calahorro, L. Méndez-Liñán, M. Pérez-Mendoza, N. Casati, E. Colacio and A. Rodríguez-Diéguez, *Chem. Commun.*, 2013, 49, 11329–11331.

55 C. X. Bezuidenhout, V. J. Smith, C. Esterhuysen and L. J. Barbour, *J Am Chem Soc*, 2017, 139, 5923–5929.

56 J. Cepeda, M. Pérez-Mendoza, A. J. Calahorro, N. Casati, J. M. Seco, M. Aragones-Anglada, P. Z. Moghadam, D. Fairen-Jimenez and A. Rodríguez-Diéguez, *J. Mater. Chem. A*, 2018, 6, 17409–17416.

57 B. Bhattacharya, R. Haldar, R. Dey, T. K. Maji and D. Ghoshal, *Dalton Trans.*, 2014, 43, 2272–2282.

58 R. Dey, R. Haldar, T. K. Maji and D. Ghoshal, *Cryst Growth Des*, 2011, 11, 3905–3911.

59 B. Chen, Y. Ji, M. Xue, F. R. Fronczek, E. J. Hurtado, J. U. Mondal, C. Liang and S. Dai, *Inorg Chem*, 2008, 47, 5543–5545.

60 N. Goel, *Inorganica Chim Acta*, 2016, 450, 330–336.

61 J. W. Uebler, A. L. Pochodylo and R. L. LaDuca, *Inorganica Chim Acta*, 2013, 405, 31–42.

62 Q.-W. Zhang, Y.-H. Wen and Y.-L. Feng, *Acta Crystallographica Section C*, 2006, 62, m255–m257.

63 H.-C. Kim, S. Mitra, M. Veerana, J.-S. Lim, H.-R. Jeong, G. Park, S. Huh, S.-J. Kim and Y. Kim, *Sci Rep*, 2019, 9, 14983.

64 H.-Y. Lin, P. Liu, X.-L. Wang, C. Xu and G.-C. Liu, 2013, 68, 138–146.

65 D. P. Martin, R. M. Supkowski and R. L. LaDuca, *Cryst Growth Des*, 2008, 8, 3518–3520.

66 Y.-Q. Zheng, J.-L. Lin and Z.-P. Kong, *Inorg Chem*, 2004, 43, 2590–2596.

67 D. N. Lee and Y. Kim, *IUCrdata*, 2017, 2, x171448.

68 X. Xu, Y. Ma and E. Wang, *J Solid State Chem*, 2007, 180, 3136–3145.

69 Y.-Q. Zheng and E.-B. Ying, *Polyhedron*, 2005, 24, 397–406.

70 R. Dey, B. Bhattacharya, P. Pachfule, R. Banerjee and D. Ghoshal, *CrystEngComm*, 2014, 16, 2305–2316.

71 G.-L. Wen, Y.-Y. Wang, W.-H. Zhang, C. Ren, R.-T. Liu and Q.-Z. Shi, *CrystEngComm*, 2010, 12, 1238–1251.

72 J. H. Nettleman, R. M. Supkowski and R. L. LaDuca, *J Solid State Chem*, 2010, 183, 291–303.

73 H.-C. Kim, S. Mitra, M. Veerana, J.-S. Lim, H.-R. Jeong, G. Park, S. Huh, S.-J. Kim and Y. Kim, *Sci Rep*, 2019, 9, 14983.

74 D. P. Martin, M. R. Montney, R. M. Supkowski and R. L. LaDuca, *Cryst Growth Des*, 2008, 8, 3091–3097.

75 J. Chi, Y. Mu, Y. Li, P. Shao, G. Liu, B. Cai, N. Xu and Y. Chen, *RSC Adv.*, 2021, 11, 31756–31765.

76 N.-Y. Li, D. Liu and J.-P. Lang, *Chem Asian J*, 2019, 14, 3635–3641.

77 A. Maiti, A. Halder, S. Dinda, G. Pahari and D. Ghoshal, *Polyhedron*, 2022, 227, 116144.

78 J. A. Wilson, J. W. Uebler and R. L. LaDuca, *CrystEngComm*, 2013, 15, 5218–5225.

79 Y.-Q. Zheng, J.-L. Lin and Z.-P. Kong, *Inorg Chem*, 2004, 43, 2590–2596.

80 N. Hao, E. Shen, Y. Li, E. Wang, C. Hu and L. Xu, *Inorg Chem Commun*, 2004, 7, 510–512.

81 H.-C. Kim, S. Huh, J. Y. Kim, H. R. Moon, D. N. Lee and Y. Kim, *CrystEngComm*, 2017, 19, 99–109.

82 X. Xu, Y. Ma and E. Wang, *J Solid State Chem*, 2007, 180, 3136–3145.

83 Y.-Q. Zheng and E.-B. Ying, *Polyhedron*, 2005, 24, 397–406.

84 R.-F. Hu, Y. Kang, J. Zhang, Z.-J. Li, Y.-Y. Qin and Y.-G. Yao, *Z Anorg Allg Chem*, 2005, 631, 3053–3057.

85 A. L. Pochodylo and R. L. LaDuca, *Inorganica Chim Acta*, 2012, 389, 191–201.

86 Y. Ding, H. Chen, E. Wang, X. Xu, X. Wang and C. Qin, *Transition Metal Chemistry*, 2008, 33, 183–187.

87 P. S. Mukherjee, S. Konar, E. Zangrando, T. Mallah, J. Ribas and N. R. Chaudhuri, *Inorg Chem*, 2003, 42, 2695–2703.

88 E. Shyu, R. M. Supkowski and R. L. LaDuca, *Cryst Growth Des*, 2009, 9, 2481–2491.

89 B. Ugale and C. M. Nagaraja, CCDC 1511819, *CSD Communication*, 2016.

90 B. Ugale and C. M. Nagaraja, CCDC 1518366, *CSD Communication*, 2016.

91 B. Ugale and C. M. Nagaraja, CCDC 1531686, *CSD Communication*, 2016.

92 M. H. Mir, L. L. Koh, G. K. Tan and J. J. Vittal, *Angewandte Chemie International Edition*, 2010, 49, 390–393.

93 G. Zhang, X. Zhou, T. Li and X. Meng, *Inorganica Chim Acta*, 2014, 421, 45–51.

94 W. X. Zhang, R. Ishikawa, B. Breedlove and M. Yamashita, *RSC Adv*, 2013, 3, 3772–3798.

95 R. A. Altman and S. L. Buchwald, *Org Lett*, 2006, 8, 2779–2782.
